# Dynamic modeling and optimal control of cystic echinococcosis

**DOI:** 10.1186/s40249-021-00807-6

**Published:** 2021-03-24

**Authors:** Xinmiao Rong, Meng Fan, Huaiping Zhu, Yaohui Zheng

**Affiliations:** 1 College of Mathematical Sciences, Harbin Engineering University, 145 Nantong Street, Harbin, Heilongjiang 150001 People’s Republic of China; 2 School of Mathematics and Statistics, Northeast Normal University, 5268 Renmin Street, Changchun, Jilin 130024 People’s Republic of China; 3 CDM, LAMPS and Department of Mathematics and Statistics, York University, 4700 Keele Street, Toronto, ON M3J 1P3 Canada; 4 Animal Health Supervision Institute of Xingan League, Tiexi North Road, Ulanhot, Inner Mongolia 137400 People’s Republic of China

**Keywords:** Cystic echinococcosis, Dynamic modeling, Optimal control, Global stability, Sheep vaccination

## Abstract

**Background:**

Cystic echinococcosis is one of the most severe helminth zoonosis with a drastic impact on human health and livestock industry. Investigating optimal control strategy and assessing the crucial factors are essential for developing countermeasures to mitigate this disease.

**Methods:**

Two compartment models were formulated to study the dynamics of cystic echinococcosis transmission, to evaluate the effectiveness of various control measures, and to find the optimal control strategy. Sensitive analyses were conducted by obtaining PRCCs and contour plot was used to evaluate the effect of key parameters on the basic reproduction number. Based on forward–backward sweep method, numerical simulations were employed to investigate effects of key factors on the transmission of cystic echinococcosis and to obtain the optimal control strategy.

**Results:**

The food resources of stray dog and invalid sheep vaccination rate, which are always neglected, were significant to the transmission and control of cystic echinococcosis. Numerical simulations suggest that, the implementation of optimal control strategy can significantly reduce the infections. Improving the cost of health education and domestic dog deworming could not decrease human infections.

**Conclusions:**

Our study showed that only a long-term use of the optimal control measures can eliminate the disease. Meanwhile, during the intervention, sheep vaccination and stray dogs disposing should be emphasized ahead of domestic dogs deworming to minimize the control cost. Simultaneously reducing other wild intermediate hosts and strengthening the sheep vaccination as well as disposing the stray dogs would be most effective.
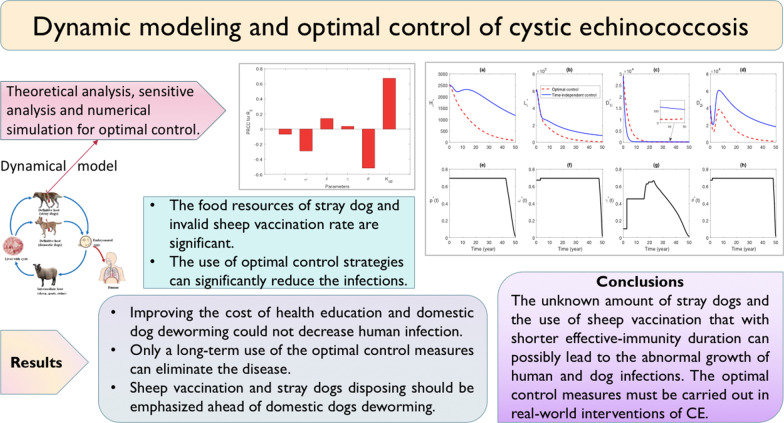

## Background

Echinococcosis is an environmental driven zoonotic disease caused by eggs of Echinococcus transmitted from carnivores, which results in substantial morbidity and mortality in most areas of the world [1]. The latest estimation for the global burden of cystic echinococcosis (CE) was 184,000 new cases per annum resulting in 184,000 disability adjusted life years (DALYs), which led to a loss of 760 million dollars a year [2, 3]. The global burden of alveolar echinococcosis (AE) was estimated to be 18,200 cases per annum, resulting in approximately 666,000 DALYs, and the mortality of infectious humans without treatment may exceed 90% in 10–15 years. Whilst, 91% of the human cases and 95% of the DALYs were assessed to be in China [4, 5]. Indeed, in western Sichuan, CE and AE have been shown to be highly endemic over 900,000 km^2^ [6]. Mass abdominal ultrasound screening result has revealed that the village prevalence rate of human CE was 12.1%, while that of human AE was 14.3% [7]. Studies of dogs showed that the dog prevalence was 14.5% [8].

**Fig. 1:**
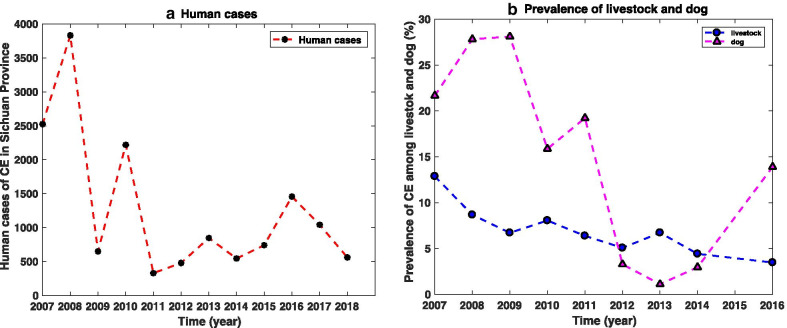
Data of human CE cases, infection ratios of sheep and dogs in Sichuan Province, China from [11–13]

Some elimination exercises were based primarily on health education, praziquantel (PZQ)-based dogs deworming or abolition of individual slaughter of sheep, most successful control cases occurred on small islands [1, 9]. As for Sichuan Province, the Ministry of Health of the People’s Republic of China launched a plan in 2006 to address the main interventions involving dog deworming, stray dogs disposing, sheep vaccination programs, and health education [10]. However, the data collected from the studies of He et al. [11, 12] and Sichuan Province [13] showed that, despite the interventions described above, the number of CE human cases fluctuates from 2007 to 2011, and then increases between 2011 and 2016 (Fig. 1a). In addition, the trend of the infection rate of dogs is similar to that of human cases, fluctuating between 2007 and 2013, and increasing significantly between 2013 and 2016 (Fig. 1b triangle line). Yet, unlike the prevalence of humans and dogs, the infection ratio of sheep keeps declining in the years from 2007 to 2016 (Fig. 1b dot line).

Actually, there are several possible factors that cause abnormalities in humans and dogs as shown in the figure. First, due to the dispersed population and seasonal problems, it is difficult to achieve a standard schedule for monthly dogs deworming and stray dogs disposing [10]. The other is that time-independent sustainability of control implementation may not be sufficient to adapt to different environments [1]. At the same time, strong CE control interventions result in high costs. Then, we want to study the impact of stray dogs on the CE transmission and explore an optimal control strategy that can reduce both CE and costs. The observations in Fig. 1b indicate that sheep control is essentially effective, but the effectiveness of the vaccination program is rarely evaluated. Hence, we will also assess the contribution of sheep vaccination to the control of this endemic.

Some mathematical models have been developed to explain the transmission dynamics among human, dog and livestock, to assess control measures and to predict intervention outcomes in some Asian countries [2, 10, 14–16]. Yang et al. [15] used statistical analysis to conclude that a control program, which combined sheep vaccination and dog anthelmintic treatment, could achieve the goal of echinococcosis control in the long term. Moss et al. [16] considered the reinfection of canine echinococcosis to investigate the role of dogs in the spread of *Echinococcus multilocularis* in Tibetan communities of Sichuan Province. The results suggested that dog deworming could be an effective strategy to reduce the endemic in those communities. Craig et al. in [10] pointed out that combining treatment and control measures to control echinococcosis was the most effective potential.

The optimal control theory is a tool to find the optimal measures among comprehensive implementation interventions, which has been applied to many control for infectious disease [17–20], including the optimal risk management of human alveolar echinococcosis in Hokkaido [21]. Usually, the control of CE remains notoriously difficult, time-consuming and costly, especially in large scale campaign in remote and larger pastoral communities [1]. The prevention and control of CE require substantial financial resources. In order to explore the mechanism of CE transmission, to investigate the optimal control strategy, and to evaluate the effectiveness of vaccination program for sheep, we formulated a new dynamic model which develops the model proposed in [22] and incorporates into the sheep vaccination program.

## Methods

### Transmission model without optimal control

**Fig. 2:**
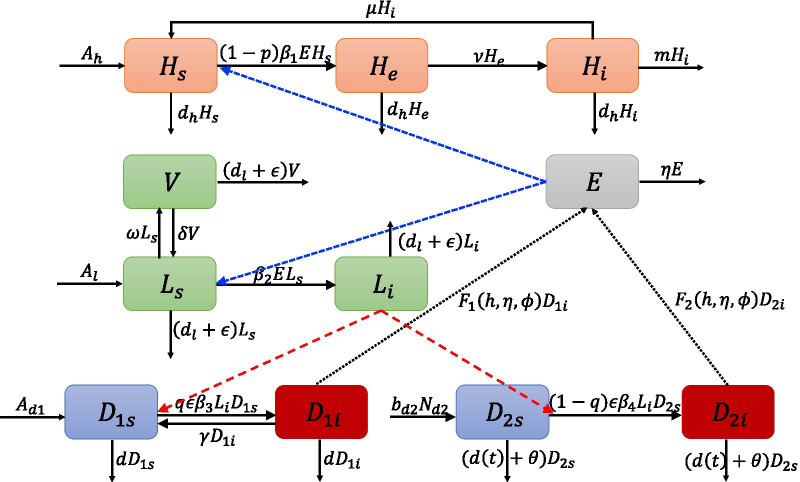
Flux diagram of the Echinococcus granulosus in human, sheep and dogs

A compartmental model is developed by adding a compartment of the intermediate host (sheep) with vaccination immunity. Our model also considers other important control measures of CE, such as health education, deworming treatment for dogs, and disposals of stray dogs. The transmission cycle of *E. granulosus* occurs through the definitive and intermediate hosts, also involves the eggs of *E. granulosus* (Fig. 2).

As in [22], we still regard the domestic dog and stray dog as different definitive hosts. The total domestic dog population $({N}_{d1}(t))$ and the total stray dog population $({N}_{d2}(t))$ are divided into two groups: the susceptible population $({D}_{1s},{D}_{2s})$ and the infectious population (*D*_1*i*_, D_2i_)$,$ then ${N}_{dj}={D}_{js}\left(t\right)+{D}_{ji}(t)$ for $j=\mathrm{1,2}$. Here, we assume that domestic dogs reproduce at a constant recruitment rate, based on the fact that the feed of domestic dogs is almost unchanged, while the excess puppies are mostly abandoned or given away [10]. Meanwhile, stray dogs are often under-fed and free to live [10], then we legitimately believe that stray dogs follow a Logistic growth due to limited food resources. Under these conditions, the growth of dogs can be expressed as following equations:

1 $$\left\{\begin{array}{l}\frac{d{N}_{d1}}{dt}={A}_{d1}-d{N}_{d1},\\ \frac{d{N}_{d2}}{dt}=\left({b}_{d2}-d\right)\left(1-\frac{{N}_{d2}}{{K}_{d2}}\right){N}_{d2}-\theta {N}_{d2},\end{array}\right.$$

where A_d1_ denotes the average annual recruitment rate of domestic dogs, d indicates the natural death rate of dogs, ${b}_{d2}$ is the birth rate of stray dogs, ${K}_{d2}$ reflects the food resources for stray dogs, and $\theta$ is the disposing rate of stray dogs. Since the stray dog keeps growing in natural setting, we assume that ${b}_{d2}>(d+\theta )$.

As intermediate hosts, the sheep becomes infected by contacting with parasitic eggs. The current vaccination program carried out in western Sichuan Province is to implement mandatory immunization for all sheep. Generally, the vaccine injection procedure consists of two parts: (1) for newborn lambs, vaccinate these lambs once at birth, and then vaccinate them again one month later; (2) for adult sheep (greater than one year of age), vaccinate them once a year [23]. Meanwhile, the vaccination program with the Eg95 vaccine has proven to be an effective intervention against the disease [24]. Correspondingly, we divide the sheep population into susceptible individuals ${L}_{s}(t)$, infectious individuals ${L}_{i}(t)$, and vaccinated individuals $V(t)$. The density of *E. granulosus* eggs depends mainly on the number of infectious domestic and stray dogs, and also relates to the mortality rate of eggs in the environment. We consider $E(t)$ as the density of *E. granulosus* eggs, which comes only from the dumping of infectious domestic and stray dogs.

Based on the flow diagram in Fig. 2 and considering human as an incidental intermediate host, we formulate the following livestock-dog-egg life-cycle model:

2 $$\left\{\begin{array}{l}\frac{d{L}_{s}}{dt}={A}_{l}-{\beta }_{2}E{L}_{s}-\omega {L}_{s}+\delta V-\left(\epsilon +{d}_{l}\right){L}_{s},\\ \frac{dV}{dt}=\omega {L}_{s}-\delta V-\left(\epsilon +{d}_{l}\right)V,\\ \frac{d{L}_{i}}{dt}={\beta }_{2}E{L}_{s}-\left(\epsilon +{d}_{l}\right){L}_{i},\\ \frac{d{D}_{1s}}{dt}={A}_{d1}-q\epsilon {\beta }_{3}{L}_{i}{D}_{1s}+\gamma {D}_{1i}-d{D}_{1s},\\ \frac{d{D}_{1i}}{dt}=q\epsilon {\beta }_{3}{L}_{i}{D}_{1s}-\gamma {D}_{1i}-d{D}_{1i},\\ \frac{d{D}_{2s}}{dt}={b}_{d2}{N}_{d2}-\left(1-q\right)\epsilon {\beta }_{4}{L}_{i}{D}_{2s}-\theta {D}_{2s}-\frac{{(b}_{d2}-d{)N}_{d2}}{{K}_{d2}}{D}_{2s}-d{D}_{2s},\\ \frac{d{D}_{2i}}{dt}=\left(1-q\right)\epsilon {\beta }_{4}{L}_{i}{D}_{2s}-\theta {D}_{2i}-\frac{\left({b}_{d2}-d\right){N}_{d2}}{{K}_{d2}}{D}_{2i}-d{D}_{2i},\\ \frac{dE}{dt}={F}_{1}\left(h,\eta ,\phi \right){D}_{1i}+{F}_{2}\left(h,\eta ,\phi \right){D}_{2i}-{\eta }_{1}E.\end{array}\right.$$

In model (2), for the sheep populations, we use ${A}_{l}$ to represent the average annual recruitment rate, $\epsilon$ is the fraction of annual slaughtered sheep, $\omega$ is the vaccination rate, $\delta$ means the invalid sheep vaccination rate $(1/\delta )$ represents the effective-immunity duration of the vaccine), ${d}_{l}$ denotes the natural death rate of sheep, ${\beta }_{2}E{L}_{s}$ describes the transmission of CE to sheep by the ingestion of E. granulosus eggs in the environment.

For the dog populations, $q$ is the livers intake fraction of domestic dogs, and $\gamma$ is the deworming recovery rate of infectious domestic dogs. In fact, sheep and cattle are still slaughtered in the traditional way and the abandoned livers are easily accessible to scavenging dogs, so the dog’s infection rate also depends on the slaughter proportion $\epsilon$. We then separate $q\epsilon {\beta }_{3}{L}_{i}{D}_{1s}$ and $(1-q)\epsilon {\beta }_{4}{L}_{i}{D}_{2s}$ as the CE transmission of domestic dogs and stray dogs after ingesting cyst-containing organs of infectious livestock.

The *E. granulosus* eggs are produced in the worms of the dog and released with its feces. We follow the assumptions in [25] and use ${F}_{k}(h,\eta ,\varphi )$ for the released rate from infectious dogs,${F}_{k} (h, \eta , \varphi ) = \varphi (h/\eta ) (1 - exp\{-\eta tk \}) (k = 1, 2)$, where $\varphi$ denotes the proportion of worms that release eggs, $h$ is the egg released rate of one worm unit time, ${t}_{k}$ describes the average lifespan of domestic and stray dogs, $\eta$ is the parasite eggs mortality rate in the dog’s small intestines, while ${\eta }_{1}$ represents the natural mortality rate of eggs in the environment. It is necessary to point out that, sheep vaccination and dog deworming are discrete intervention strategies, in this study, all intervention are assumed to be continuous deployment since the time scale here is selected as ‘year’.

Furthermore, in order to raise public awareness, some moderate measures have been taken to protect against CE such as human health education. We assume that human infection is linearly dependent on the amount of released eggs, and then we use $\left(1-p\right){\beta }_{1}E{H}_{s}$ as the CE infection rate. When humans are infected by eggs, humans may take months or even years to show symptoms [26], we introduce the incubation period of infected (exposed) individuals, represented by $1/\nu$. When a cyst in the human body is excised, the infectious individual recovers from the infection as recovery rate $\mu$. The total human population is separated into the classes of susceptible ${H}_{s}(t),$ exposed ${H}_{e}(t),$ and infectious ${H}_{i}(t)$. We have the following equations for human

3 $$\left\{\begin{array}{l}\frac{d{H}_{s}}{dt}={A}_{h}-\left(1-p\right){\beta }_{1}E{H}_{s}+\mu {H}_{i}-{d}_{h}{H}_{s},\\ \frac{d{H}_{e}}{dt}=\left(1-p\right){\beta }_{1}E{H}_{s}-\nu {H}_{e}-{d}_{h}{H}_{e},\\ \frac{d{H}_{i}}{dt}=\nu {H}_{e}-\left(\mu +{d}_{h}+m\right){H}_{i},\end{array}\right.$$

where ${A}_{h}$ is the constant recruitment rate of human population, $p$ denotes the influence coefficient of publicity measures, m and ${d}_{h}$ are the disease induced and natural death rate, respectively. Here all parameters are positive and their biological significance, default values, and reference resource are summarized in Table 1. Systems (2) and (3) are coupled, we will treat them together for the purpose of control and prevention of CE.

**Table 1:** Parameters of model (0.2) and (0.3) with default values

Parameters	Biological definition	Value (range)	Source
${A}_{h}$	Annual recruitment rate of human population	$7.482\times {10}^{4}$	[35]
$p$	Influence coefficient of publicity measures	0.1 (0–1)	Set
$\mu$	Recovery rate of humans who are received surgery	0.75	[13]
${d}_{h}$	Natural death rate of humans	1/76.5	[35]
$m$	The mortality rate induced by CE	0.022	[36]
${\beta }_{1}$	Prevailing infection pressure between parasite eggs and humans	$1.004\times {10}^{-12}$	Esti
$\nu$	Transition rate from exposed humans to infectious humans	1/14	[13]
${A}_{l}$	Annual recruitment rate of sheep	$6.88\times {10}^{5}$	[35]
${\beta }_{2}$	Prevailing infection pressure between parasite eggs and sheep	$1.08\times {10}^{-9}$	Esti
$\epsilon$	Fraction of annual slaughtered sheep	0.2 (0.16–0.667)	Assumption
$\omega$	Vaccination rate of sheep	0.4 (0.2–1)	Set
$\delta$	Invalid sheep vaccination rate	0.67 (0.5–2)	Assumption
${d}_{l}$	Natural death rate of sheep	0.152	[35]
${A}_{d1}$	Annual recruitment rate of domestic dogs	$2\times {10}^{5}$	[35]
$q$	The proportion of domestic dogs intake the livers of sheep	0.3 (0–1)	Set
${\beta }_{3}$	Prevailing infection pressure between infectious sheep and domestic dogs	$7.1\times {10}^{-9}$	Esti
$\gamma$	Deworming recovery rate of infectious domestic dogs	0.4 (0.1–0.9)	Set
$d$	Natural death rate of dog	0.08	[37]
${b}_{d2}$	Birth rate of stray dog	2	Assumption
${K}_{d2}$	The food resources of stray dogs in the environment	8.5 × 10^7^ (6.5–10.5) × 10^7^	Assumption
${\beta }_{4}$	Prevailing infection pressure between infectious sheep and stray dogs	$5\times {10}^{-8}$	Esti
$\theta$	Disposing rate of stray dogs	0.4 (0.1–0.9)	Set
$\phi$	Egg released rate of one worm unit time	42	[38]
$h$	Worm produced rate per dog per year	560	[14]
$\eta$	Parasite eggs mortality rate in the dogs body	12/5	[14]
${t}_{1} ({t}_{2})$	The average lifespan of domestic dogs (stray dogs)	6 (4)	[14]
${\eta }_{1}$	Parasite eggs natural mortality in the environment	10.42	[37]

### Transmission model with optimal control

The CE asserts a heavy burden to human health and the socio-economics, while the government has limited financial support for control and prevention against CE. So, endemic-level optimal control measures must be carefully assessed. To determine the optimal control strategy, we reformulate our model (2) and (3) to include time-dependent anti-CE control measures.

Since the CE cases of human and infectious rate of dogs are still fluctuating even under the implementation of existing time-independent control measures (see Fig. 1), it is more reasonable to develop the optimal control strategy involving time-dependent control measures. Then the publicity measure $p$ is set to be time-dependent $p(t)$ to describe the time-varying health education strategy. One other important step is the sheep vaccination program, we will use $\omega (t)$ to measure the reduction rate due to the sheep vaccine protection. Furthermore, the recovery rate of infectious domestic dogs under the vermicide $\gamma (t)$ and the disposing rate of stray dogs $\theta (t)$ represent the control efforts on domestic dogs and stray dogs, respectively.

Then, we modify model (2) and (3) as the following

4 $$\left\{\begin{array}{l}\frac{d{H}_{s}}{dt}={A}_{h}-\left(1-p\left(t\right)\right){\beta }_{1}E{H}_{s}+\mu {H}_{i}-{d}_{h}{H}_{s},\\ \frac{d{H}_{e}}{dt}=\left(1-p\left(t\right)\right){\beta }_{1}E{H}_{s}-\nu {H}_{e}-{d}_{h}{H}_{e},\\ \frac{d{H}_{i}}{dt}=\nu {H}_{e}-\left(\mu +{d}_{h}+m\right){H}_{i},\\ \frac{d{L}_{s}}{dt}={A}_{l}-{\beta }_{2}E{L}_{s}-\omega \left(t\right){L}_{s}+\delta V-\left(\epsilon +{d}_{l}\right){L}_{s},\\ \frac{dV}{dt}=\omega \left(t\right){L}_{s}-\delta V-\left(\epsilon +{d}_{l}\right)V,\\ \frac{d{L}_{i}}{dt}={\beta }_{2}E{L}_{s}-\left(\epsilon +{d}_{l}\right){L}_{i},\\ \frac{d{D}_{1s}}{dt}={A}_{d1}-q\epsilon {\beta }_{3}{L}_{i}{D}_{1s}+\gamma \left(t\right){D}_{1i}-d{D}_{1s},\\ \frac{d{D}_{1i}}{dt}=q\epsilon {\beta }_{3}{L}_{i}{D}_{1s}-\gamma \left(t\right){D}_{1i}-d{D}_{1i},\\ \frac{d{D}_{2s}}{dt}={b}_{d2}{N}_{d2}-\left(1-q\right)\epsilon {\beta }_{4}{L}_{i}{D}_{2s}-\theta \left(t\right){D}_{2s}-\frac{{(b}_{d2}-d{)N}_{d2}}{{K}_{d2}}{D}_{2s}-d{D}_{2s},\\ \frac{d{D}_{2i}}{dt}=\left(1-q\right)\epsilon {\beta }_{4}{L}_{i}{D}_{2s}-\theta \left(t\right){D}_{2i}-\frac{\left({b}_{d2}-d\right){N}_{d2}}{{K}_{d2}}{D}_{2i}-d{D}_{2i},\\ \frac{dE}{dt}={F}_{1}\left(h,\eta ,\phi \right){D}_{1i}+{F}_{2}\left(h,\eta ,\phi \right){D}_{2i}-{\eta }_{1}E.\end{array}\right.$$

Here, our goal is to identify an integrated control strategy that jointly minimizes the number of infectious human as well as sheep population and the cost of control programs. Mathematically, using the system (4), we have developed an optimal control problem with the objective functional defined as

5 $$J\left(p\left(t\right),\omega \left(t\right),\gamma \left(t\right), \theta \left(t\right)\right) =\underset{0}{\overset{T}{\int }}({B}_{0}{H}_{i}(t) +{B}_{1} {L}_{i}(t)+{C}_{1}{p}^{2}(t)+{C}_{2}{\omega }^{2}\left(t\right)+{C}_{3}{\gamma }^{2}\left(t\right) +{C}_{4}{\theta }^{2}(t))dt$$

where $B_{0}$ and $B_{1}$ represent, respectively, the weight constants of the infectious human and infected sheep populations. ${C}_{1},{C}_{2},{C}_{3},{C}_{4}$ are balancing coefficients transforming the integral into cost expended over a finite time period of T years. The terms ${C}_{1}{p}^{2}(t)$ and ${C}_{2}{\omega }^{2}(t)$ describe the total costs associated with human population’s health education and vaccination of whole sheep population, respectively. ${C}_{3}{\gamma }^{2}(t)$ and ${C}_{4}{\theta }^{2}(t)$ read the costs for anthelmintic control of domestic dogs and disposing of stray dogs. Here the objective functional $J(p(t),\omega (t),\gamma (t),\theta (t))$ measures the total economic loss caused by CE. That is, we need to determine an optimal control $({p}^{*}\left(t\right),{ \omega }^{*}\left(t\right),{ \gamma }^{*}\left(t\right), {\theta }^{*}(t))$ such that

6 $$J\left(\left\{{p}^{*}\left(t\right),{\omega }^{*}\left(t\right),{\gamma }^{*}\left(t\right),{\theta }^{*}\left(t\right)\right\}\right)=\underset{U}{\mathrm{min} }(\{p(t),\omega (t),\gamma (t),\theta (t)\}),$$

where $U = \{(p(t),\omega (t),\gamma (t),\theta (t))| s.t. 0 \le p(t) \le {p}_{max},0 \le \omega (t) \le {\omega }_{max},0 \le \gamma (t) \le {\gamma }_{max}, 0 \le \theta (t) \le {\theta }_{max}, t \in [0, T ]\}$ is the feasible decision space or the control set, which is closed and convex.

Note that the integrand of the objective functional given by (5) is convex on $U$, the model is linear in the control variables and is bounded by a linear system in the state variables. By Theorem 4.1 and Corollary 4.1 in [27], there exists an optimal control $({p}^{*}\left(t\right),{ \omega }^{*}\left(t\right),{ \gamma }^{*}\left(t\right), {\theta }^{*}(t))$, such that (6) holds, and the state solution corresponding to the optimal control reads $H{s}^{*}\left(t\right), H{e}^{*}\left(t\right), H{i}^{*}\left(t\right), {L}_{s}^{*}\left(t\right), {V}^{*}\left(t\right), {L}_{i}^{*}\left(t\right), {D}_{1s}^{*}\left(t\right), {D}_{1i}^{*}\left(t\right), {D}_{2s}^{*}\left(t\right), { D}_{2i}^{*}(t), { E}^{*}(t)$, find more details in the Additional file 1.

### Parameter values setting

The values of parameters involved in the models (2) and (3) were obtained from references and estimation, see Table 1. For simulations of the model (4), based on the official survey reports and personal communication with professionals, we took the weights ${B}_{2} = \mathrm{15,000}$ and ${B}_{1} = 300$, which means that more effort is given to the minimization of the infectious humans than that to the reduction of infected sheep. We set $p = 0.1, \omega = 0.3, \gamma = 0.65$ and $\theta = 0.43$ due to the current control status in western Sichuan Province. Meanwhile, from the survey report [28], the cost for health education in Sichuan was about 300,000 RMB per year and the cost for sheep vaccination and the treatment of infectious disease was about 896,000 RMB per year. We then assumed that the total cost for each control measure is same, i.e., ${C}_{1} = {C}_{2} ={C}_{3}={C}_{4}= \mathrm{300,000}$. Since the controls would not be 100% effective, without any loss of generality, we assumed that the upper bounds of the four control measures are set to be 0.7, i.e., ${p}_{max }={\omega }_{max}={\gamma }_{max}= {\theta }_{max}= 0.7$.

### Sensitivity analyses and evaluation of optimal control strategy

Sensitivity analyses were conducted by evaluating the partial rank correlation coefficients (i.e., PRCCs) for various parameters against the basic reproduction number ${R}_{0}$ over time. The parameters considered here were control measure $(\omega , \gamma , \theta )$, annual sheep slaughtered fraction $(\varepsilon )$, invalid sheep vaccination rate $(\delta )$, and food resources of stray dogs $({K}_{d2})$. Contour plot for ${R}_{0}$ was used to evaluate the effect of $\delta$ and ${K}_{d2}$ on disease prevalence and control outcomes. Furthermore, by using “forward–backward sweep method” [29], we numerically simulated the implementation of optimal control strategy for significant reduction of infections of human $({H}_{i}),$ sheep $({L}_{i}),$ domestic dog $({D}_{1i})$, and stray dog $({D}_{2i})$.

## Results

### Theoretical results

Obviously, the system always has a trivial equilibrium ${E}_{df0}$ and a disease-free equilibrium (DFE) ${E}_{dfe}$, which reads

$${E}_{df0}=\left({H}_{s}^{0},\mathrm{0,0},{L}_{s}^{0},{V}^{0},0,{D}_{1s}^{0},\mathrm{0,0},\mathrm{0,0}\right), {E}_{dfe}=\left({H}_{s}^{0},\mathrm{0,0},{L}_{s}^{0},{V}^{0},0,{D}_{1s}^{0},0,{D}_{2s}^{0},\mathrm{0,0}\right),$$

where $${H}_{s}^{0}={A}_{h}/{d}_{h}$$, $${L}_{s}^{0}=\alpha {A}_{1}/({d}_{l}+\epsilon )$$, $${V}^{0}=\left(1-\alpha \right){A}_{l}/({d}_{l}+\epsilon )$$, $${D}_{1s}^{0}={A}_{d1}/d$$, $${D}_{2s}^{0}=\left({b}_{d2}-d-\theta \right){K}_{d2}/({b}_{d2}-d)$$, and $$\alpha =(\delta +\epsilon +{d}_{l})/(\omega +\delta +\epsilon +{d}_{l})$$.

Following the next generation matrix method developed by [30], the new infection terms and the remaining transfer terms are given respectively by $F$ and $V$, and the basic reproduction number ${R}_{0}$, which is calculated from $\rho (F{V}^{-1})$ (see the Additional file 1), is given by

7 $$\sqrt[3]{\frac{{\beta }_{2}{\stackrel{-}{\beta }}_{3}\alpha {A}_{l}{A}_{d1}{F}_{1}}{{\left(\epsilon +{d}_{l}\right)}^{2}\left(\gamma +d\right)d{\eta }_{1}}+\frac{{\beta }_{2}{\stackrel{-}{\beta }}_{4}\alpha {A}_{l}\left({b}_{d2}-d-\theta \right){K}_{d2}{F}_{2}}{{\left(\epsilon +{d}_{l}\right)}^{2}\left({b}_{d2}-d\right){b}_{d2}{\eta }_{1}}=\sqrt[3]{{R}_{10}^{3}+{R}_{20}^{3}} } ,$$

where $${\stackrel{-}{\beta }}_{3}=q\epsilon {\beta }_{3}$$, $${\stackrel{-}{\beta }}_{4}=(1-q)\epsilon {\beta }_{4}$$, $${F}_{k}={F}_{k}\left(h,\eta ,\phi \right), k=\mathrm{1,2}$$ and

$$R_{10} = \sqrt[3]{{\underbrace {{F_{1} /\eta_{1} }}_{eggs\;by\;per\;domestic\;dog} \cdot \underbrace {{(\beta_{2} \alpha A_{l} )/[\left( {\gamma + d} \right)\left( {\epsilon + d_{l} } \right)]}}_{infected\;sheep\;by\;eggs} \cdot \underbrace {{\left( {\overline{\beta }_{3} A_{d1} } \right)/\left[ {\left( {\epsilon + d_{l} } \right)d} \right]}}_{infected\;domestic\;dogs}}}$$

$$R_{20} = \sqrt[3]{{\underbrace {{F_{2} /\eta_{1} }}_{eggs\;by\;per\;stray\;dog} \cdot \underbrace {{(\beta_{2} \alpha A_{l} )/[b_{d2} \left( {\epsilon + d_{l} } \right)]}}_{infected\;sheep\;by\;eggs} \cdot \underbrace {{(\overline{\beta }_{4} K_{d2} \left( {b_{d2} - d - \theta } \right)/\left[ {\left( {\epsilon + d_{l} } \right)\left( {b_{d2} - d} \right)} \right]}}_{infected\;stray\;dogs}}}$$

The basic reproduction number ${R}_{0}$ is a key indicator measuring the average new infections produced by infectious dogs as presented in [31]. ${R}_{10}$ is the average number of secondary infectious individuals (domestic dogs) only generated by an infectious domestic dogs [30]. Similarly, ${R}_{20}$ is the average number of the infectious individuals (stray dogs) which infected only by stray dogs. The cube root measures the infection power of CE into three-step transmission cycle, from infectious dogs to E. granulosus eggs, to sheep, and then to infectious dogs.

We obtained the existence of the domestic dog-drive endemic equilibrium and the endemic equilibrium through solving the systems (2) and (3) for ${R}_{10}>1$ and ${R}_{0}>1$, respectively. In addition, we theoretically proved that the trivial and domestic dog-drive endemic equilibria are always unstable, the disease-free equilibrium is locally asymptotically stable if ${R}_{0}<1$ and further it is globally asymptotically stable when ${R}_{0}<1$. Furthermore, the global stability of the endemic equilibrium proved by the methods in [32, 33, 39–41]. The results are established in the Additional file 1.

### Simulation results

#### Sensitive analysis and evaluation of key parameters

**Fig. 3:**
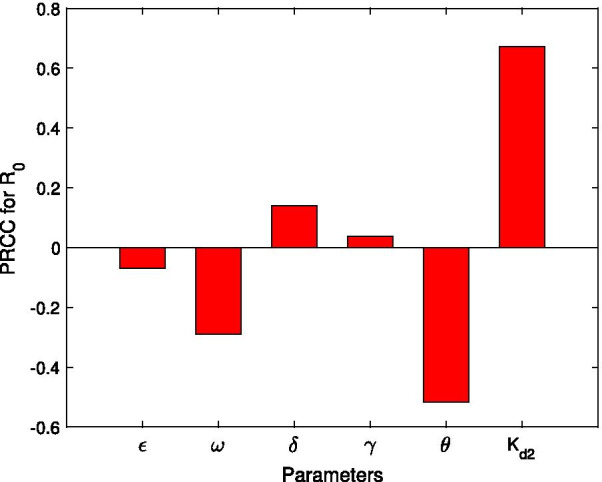
Sensitive analysis of the basic reproduction number $R_{0}$ via parameters

**Fig. 4:**
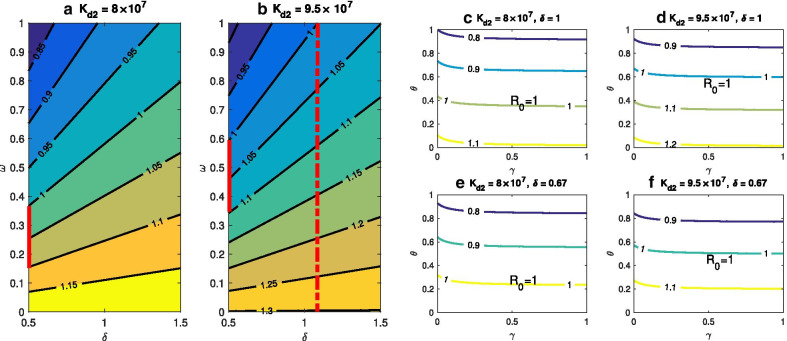
Contour plots of $R_{0}$. **a**, **b** the $(\delta ,\omega )$ planes with various stray dogs’ food resources $K_{d2}$. The contour plots measure the effectiveness of invalid sheep vaccination rate $(\delta )$ and vaccination rate $(\omega )$ on reducing $R_{0}$. **c**–**f** the $(\gamma ,\theta )$ planes respond to different $K_{d2}$ as well as $\delta$. The contour plots depict the effectiveness of domestic dog’s recovery rate $(\gamma )$ and stray dog’s disposing rate $(\theta )$ on decreasing $R_{0}$. Other parameters are listed in the Table 1

The result illustrated in Fig. 3 suggests that, for the chosen ‘control parameter’ ranges, ${R}_{0}$ is more sensitive to $\theta$ and $\omega$, followed by $\gamma$. In particular, it should be noted that ${K}_{d2}$ and $\delta$ show a significant impact on ${R}_{0}$ (the values of the PRCCs for ${K}_{d2}$ and $\delta$ are big), while these two factors are always neglected. In addition, these results indicate that both the administration and living resources of stray dogs contribute more to the transmission of CE. The analyses are demonstrated in Fig. 4.

The contour plots of ${R}_{0}$ responding to the invalid sheep vaccination rate $(\delta )$ and the vaccination rate $(\omega )$ for different stray dog resources $({K}_{d2})$ are showed in Fig. 4a, b. It indicates that, when the stray dog’s food become richer, in order to guarantee ${R}_{0}<1$, we must decrease the invalid sheep vaccination rate and increase the vaccination rate. For example, when ${K}_{d2}=8\times {10}^{7}$, the minimum of $\omega$ such that ${R}_{0}<1$ is 0.38, while when $\omega \ge 0.8$, ${R}_{0}<1$ for any $\delta$ even if $\delta$ reaches the maximum value 1.5 (Fig. 4a; when ${K}_{d2}=9.5 \times {10}^{7}$, in order to have ${R}_{0}<1$ the minimum of $\omega$ now is 0.6 and $\delta$ must be less than 1.1 (see the dashed line in Fig. 4b). In addition, Fig. 4a, b show that, in order to achieve the same effectiveness of vaccination, it is more difficult to reduce $R0$ from 1.1 to 1 when ${K}_{d2}=9.5 \times {10}^{7}$ (e.g., for $\delta =0.5$ in (a)-(b), the solid line in Fig. 4b is longer).

The joint effects of γ and θ on decreasing ${R}_{0}$ for ${K}_{d2}=8\times {10}^{7}$ or $9.5\times {10}^{7}$, $\delta = 1$ or 0.67 are illustrated in Fig. 4c–f, respectively. We observe that ${R}_{0}$ is more sensitive to θ than γ in all four cases. When ${K}_{d2}$ or $\delta$ is bigger, in order to reduce ${R}_{0}$ such that ${R}_{0}<1$, one has to carry out intensive control of dogs (especially stray dogs). It is also observed that, for more food resources of stray dogs and higher invalid sheep vaccination rate, it is much more difficult to reduce ${R}_{0}$ such that ${R}_{0}<1$ (e.g., in Fig. 4d, ${R}_{0}<1$ only when $\theta > 0.6$). In general, more food resources of stray dog and higher invalid sheep vaccination rate may have more negative effect on the CE control.

### Advantage of optimal control

**Fig. 5:**
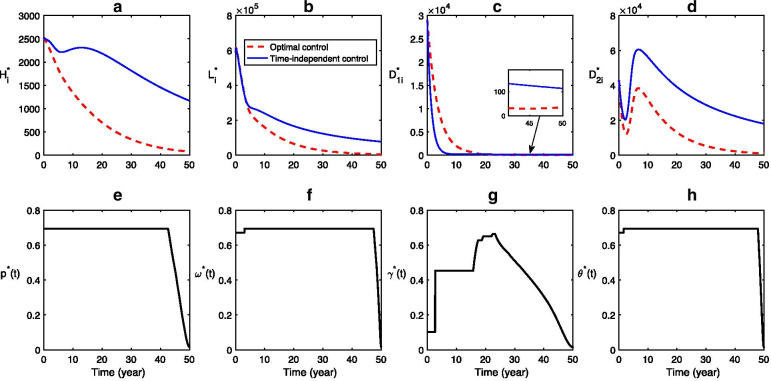
Advantage of optimal control. **a**–**d** Time series of the infections corresponding to the time-independent control (solid curves) and the optimal control (dashed curves). **e**–**h** The optimal control strategies for health education $({p}^{*}(t))$, sheep vaccination $({\omega }^{*}(t))$, domestic dog deworming $({\gamma }^{*}(t))$ stray dog disposing $({\theta }^{*}(t))$. Here C_1_ = C_2_ = C_3_ = C_4_ = 3 × 10^5^, other parameter values are listed in Table 1, and the initial conditions are ${H}_{s}\left(0\right)=4 580 000, {H}_{e}\left(0\right)=26 058, {H}_{i}\left(0\right)=2525, {L}_{s}\left(0\right)=1 958 000, V\left(0\right)=2 038 000, {L}_{i}\left(0\right)=590 321, {D}_{1s}\left(0\right)=96 900, {D}_{1i}\left(0\right)=29 090, {D}_{2s}\left(0\right)=145 350, {D}_{2i}\left(0\right)=42 892, E\left(0\right)=271 400 000.$ In the time-independent control, $p = 0.1, \omega = 0.3, \gamma = 0.65$ and $\theta = 0.43.$

The time-series dynamical scenarios of infectious hosts are performed for the system with time-independent control (solid curves in Fig. 5(a-d)) and with the optimal control efforts (dashed curves in Fig. 5(a-d)), respectively. Figure 5(e–h) illustrates the optimal control strategy, which is obtained by the forward–backward sweep Runge- Kutta method [28]. It is observed that, in Fig. 5(a,b,d), the dash curve is always below the solid curve, while, in Fig. 5(c), the dash curve is above the solid curve first and then falls below the solid curve. These facts imply that the time-independent controls cannot effectively control CE transmission, while the optimal control strategy is more effective than the time-independent control, which can significantly reduce the infections and can eliminate infections of all hosts eventually. In order to minimize both the CE infections and the economic losses, in the optimal control strategy, the health education, sheep vaccination, and stray dog disposal are supposed to be kept at the maximum level for most time and finally decrease sharply (Fig. 5e, f, h); due to the less amount of domestic dogs, the domestic dog deworming increases first to its maximum and then decreases Fig. 5g rather than keeps at a constant high level in the time-independent control (i.e., $\gamma =0.65$). The number of infectious domestic dogs corresponding to the optimal control is still less than that corresponding to the time-independent control (Fig. 5c). It implies again that the optimal control strategy is more realistic and effective.

### Effect of control cost

In the following numerical experiments, in order to further characterize the optimal control strategy by investigating the effect of different control costs ${C}_{i} (i=1, 2, 3, 4)$ on the effectiveness of optimal control, we took ${C}_{i}=3\times {10}^{5}$ or $3\times {10}^{6}$, $i=1, 2, 3, 4$, and leave other parameters ${C}_{j}=3\times {10}^{5}, j\ne i, j=1, 2, 3, 4.$

**Fig. 6:**
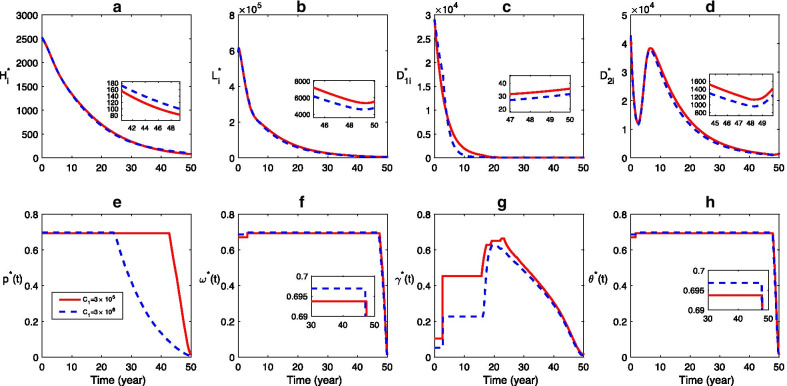
Effect of control cost for health education: **a**–**d** Time series dynamics of infections, **e**–**h** The optimal control strategy. The solid and dashed curves represent the scenarios for ${C}_{1}=3\times {10}^{5}$ and ${C}_{1}=3\times {10}^{6}$, respectively. Here ${{C}_{2}=C}_{3}={C}_{4}=3\times {10}^{5}$, the values of other parameters are listed in Table 1, and the initial conditions are same with those in Fig. 5

Compared to the scenarios with lower cost (${C}_{1}=3\times {10}^{5}$), when the cost of health education is higher ($C_{1} = 3 \times {10}^{6}$), the final size (number of infection at the end of control period, i.e., $t=50$) of human infection is slightly higher while the final sizes of both sheep and dog infections are lower (Fig. 6). The reason is that the control effort for human health education and domestic dog deworming decreases due to the higher cost of health education, while the sheep vaccination and stray dog disposal increase (Fig. 6e–h). Moreover, although the effort for domestic dog deworming decreases when ${C}_{1}=3\times {10}^{6}$, the final infection size of domestic dogs becomes lower (Fig. 6c).

**Fig. 7:**
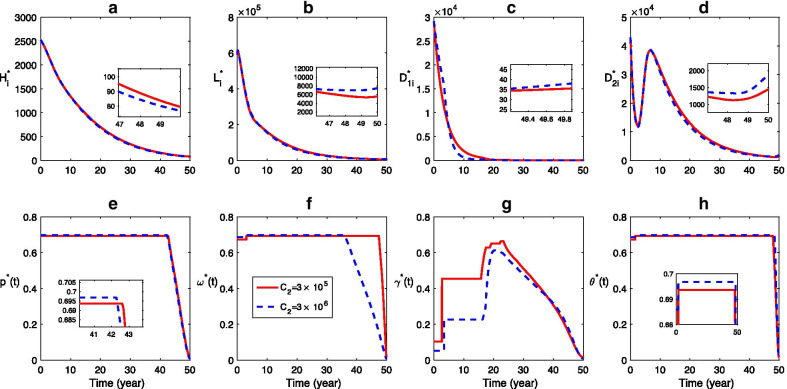
Effect of control cost for sheep vaccination: **a**–**d** Time series dynamics of infections, **e**–**h** The optimal control strategy. The solid and dashed curves correspond to the scenarios for ${C}_{2}=3\times {10}^{5}$ and ${C}_{2}=3\times {10}^{6}$, respectively. Here ${{C}_{1}=C}_{3}={C}_{4}=3\times {10}^{5}$, the values of other parameters and the initial conditions are same with those in Fig. 6

**Fig. 8:**
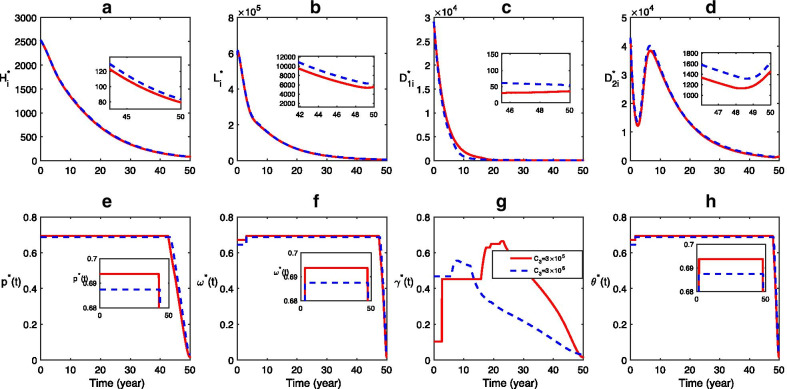
Effect of control cost for domestic dog deworming: **a**–**d** Time series dynamics of infections, **e**–**h** The optimal control strategy. The solid and dashed curves correspond to the scenarios for ${C}_{3}=3\times {10}^{5}$ and ${C}_{3}=3\times {10}^{6}$, respectively. Here ${C}_{1}={C}_{2}={C}_{4}=3\times {10}^{5}$, the values of other parameters and the initial conditions are same with those in Fig. 6

**Fig. 9:**
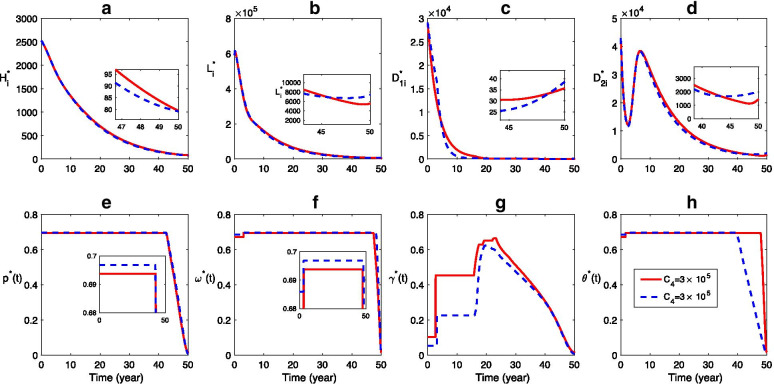
Effect of control cost for stray dog disposing: **a**–**d** Time series dynamics of infections, **e**–**h** The optimal control strategy. The solid and dashed curves correspond to the scenarios for ${C}_{4}=3\times {10}^{5}$ and ${C}_{4}=3\times {10}^{6}$, respectively. Here ${c}_{1}={C}_{2}={C}_{3}=3\times {10}^{5}$, the values of other parameters and the initial conditions are same with those in Fig. 6

Similarly, we investigate the effects of different costs of sheep vaccination, domestic dog deworming, and stray dog disposing on the CE control (Figs. 7, 8, 9). Figure 7 shows that, higher cost of sheep vaccination leads to less control effort for sheep vaccination, domestic dog deworming, and stray dog disposing but more effort for long-term human health education (Fig. 7e–h). Due to the variation of those controls, the final size of human cases declines lightly while the final size of other infections do not increase much comparing to the case with lower cost of sheep vaccination (Fig. 7a–d). The increase of cost for domestic dog deworming reduces the effort for all the four control measures (Fig. 8e–h), which is followed by an increase of the final size in all infections (Fig. 8a–d). In addition, when the cost for stray dog control is higher, the final size of human cases decreases while the final sizes of other host infections become higher than those of the situation with lower cost of stray dog disposal (Fig. 9).

## Discussion

The transmission of CE has been a growing public concern in China. Particularly, despite the effort of public health personals to strictly enforce the control measures proposed in 2006, the CE prevalent scenarios in western Sichuan Province for both human and dog exhibit an abnormal upward trend between 2007 and 2016 (see Fig. 1). Several possible factors that resulted in this unexpected trend include the effects of time-independent control, the effectiveness of sheep vaccination, and stray dog activities [1, 10, 24]. Therefore, in addition to examining the transmission mechanism, we also explore effects of two key factors (${K}_{d2}$ and $\delta$) on the CE transmission and the optimal control strategy. The model developed in this study extends the model in [22] by containing vaccinated sheep population and considering the crowding effect of stray dogs. The inclusion of vaccinated sheep immunity and stray dog’s logistic-growth assumption provides a more realistic illustration of CE transmission in western Sichuan Province.

Both analytical and simulation results suggest the importance of the food resources of stray dogs and the invalid sheep vaccination rate in determining the control outcomes. As shown in 3 and Fig. 4, the abundance of stray dog’s food resources restrains the elimination of CE (i.e., make it more difficult to reduce ${R}_{0}$ less than 1), which is consistent with the result in [22]. The simulation results for optimal control show that the optimal controls are capable of reducing all infections to zero, and is more effective than the time-independent controls (Fig. 5). Note that, to minimize both the infections and control costs, the control of sheep and stray dogs should maintain at a high level throughout the intervention, while the control efforts of domestic dogs can be reduced in the later stage to save resources.

Furthermore, the scenarios shown in Figs. 6, 7, 8, 9 indicate that, the different costs for these three control measures also contribute mildly to the reduction of all infections. The higher cost of human education could lead to a higher final size of human infections (see (a)–(d) in Fig. 6). It suggests that increasing the cost of health education could not help to control human CE, but possibly reduce the final size of infection of sheep, domestic dog and stray dog. Hence, it is ineffective to reduce human infection by enhancing the financial effort for human health education. While the greater control cost of sheep and stray dog can make the final size of human infections be smaller, and the higher control cost of domestic dog deworming will result in a larger final size of all infections. Hence, sheep vaccination and stray dog disposal are more effective to control human CE infection, which is consistent with the results of sensitive analysis. Therefore, in order to control CE in all hosts, it is reasonable and acceptable to emphasize more on sheep vaccination and control of stray dogs, and then reduce the financial effort of the domestic dog deworming.

Although the numerical simulations show that CE may be effectively controlled by adopting the control strategies, the values of weight coefficients in the objective functional are usually subjectively determined. Note that, to apply the tools developed here, one would need to know estimates of actual costs and upper bounds on the controls. The roadmap of elimination programme of echinococcosis has been defined by Chinese government. Currently, there is a 10 year timeframe to reach the goal of “Healthy China 2030” [34]. The elimination of Echinococcosis by 2030 is an urgent task. In most portfolios studies, cost is one of the major factors to determine the successful strategy. Comprehensive consideration including the political may has higher impact than financing cost, then, sufficient resources will be employed to the Echinococcosis elimination. In addition, efficacy of treatment, vaccine, or deworming is not equal to community effectiveness. Many other significant factors, such as access rate, targeting accuracy, provider compliance, consumer adherence rate, and so on, all affect the final real control effects. For example, when carrying on deworming programme, dogs will recover partially but may be with very high re-infection rate. Also due to deworming programme, large quantity of eggs will be discharged into environment and consequently may cause many new infection to human and other hosts if the dog’s droppings are not properly disposed. Investigating how these factors affect the control and elimination of Echinococcosis is essential for developing more successful strategies. Therefore, it is reasonable and realistic to explore what control strategies can be used to eliminate Echinococcosis in a shortest time (e.g., Before 2030). We leave this topic for the future investigation.

## Conclusions

Our findings suggest that, the unknown amount of stray dogs and the use of sheep vaccination that with shorter effective-immunity duration can possibly lead to the abnormal growth of human and dog infections. Therefore, the optimal control measures must be carried out in real-world interventions of CE. It is reasonable and realistic to improve the effectiveness of vaccination and to reduce the food resources of stray dog, e.g., one can increase the effort for the pre-test of sheep vaccination and reduce the richness of other wild intermediate hosts such as voles, *Ochotona curzoniaes*.

## Supplementary Information

**Additional file 1:** Theoretical analyses.

## Data Availability

The data that support the findings of this study are available from the work [11, 12] and Sichuan Center for Disease Control and Prevention (https://www.sccdc.cn/View.aspx?id=012003).
