# Supplementary material for: Dynamic modeling and optimal control of cystic echinococcosis
Source: Infect Dis Poverty. 2021 Mar 24;10:38. doi: 10.1186/s40249-021-00807-6 (PMC7992812; doi:10.1186/s40249-021-00807-6)
Supplement: Supplementary file 1 — Additional file 1: Theoretical analyses. [file 40249_2021_807_MOESM1_ESM.docx]

**Appendix**

In order to facilitate readers, we rewrite the systems of differential equations de- scribing our model with invariable control and time-dependent control, and the objective functional as following

1. The livestock-dog-egg life-cycle model

$$\left\{ \begin{aligned} &\frac{dL_{s}}{dt}=A_{l}-\beta_{2}EL_{s}-\omega L_{s}+\delta V-\left( \epsilon+d_{l} \right)L_{s}, \\ &\frac{dV}{dt}=\omega L_{s}-\delta V-\left( \epsilon+d_{l} \right)V, \\ &\frac{dL_{i}}{dt}=\beta_{2}EL_{s}-\left( \epsilon+d_{l} \right)L_{i}, \\ &\frac{dD_{1s}}{dt}=A_{d1}-q\epsilon\beta_{3}L_{i}D_{1s}+\gamma D_{1i}-dD_{1s}, \\ &\frac{dD_{1i}}{dt}=q\epsilon\beta_{3}L_{i}D_{1s}-\gamma D_{1i}-dD_{1i}, \\ &\frac{dD_{2s}}{dt}=b_{d2}N_{d2}-\left( 1-q \right)\epsilon\beta_{4}L_{i}D_{2s}-\theta D_{2s}-\frac{{(b}_{d2}-d{)N}_{d2}}{K_{d2}}D_{2s}-dD_{2s}, \\ &\frac{dD_{2i}}{dt}=\left( 1-q \right)\epsilon\beta_{4}L_{i}D_{2s}-\theta D_{2i}-\frac{\left( b_{d2}-d \right)N_{d2}}{K_{d2}}D_{2i}-dD_{2i}, \\ &\frac{dE}{dt}=F_{1}\left( h,\eta,\phi\right)D_{1i}+F_{2}\left( h,\eta,\phi\right)D_{2i}-\eta_{1}E. \end{aligned} \right.$$

$$\left( 0.1 \right)$$

2. The transmission model of the total human population

$$\left\{ \begin{aligned} &\frac{dH_{s}}{dt}=A_{h}-\left( 1-p \right)\beta_{1}EH_{s}+\mu H_{i}-d_{h}H_{s}, \\ &\frac{dH_{e}}{dt}=\left( 1-p \right)\beta_{1}EH_{s}-\nu H_{e}-d_{h}H_{e}, \\ &\frac{dH_{i}}{dt}=\nu H_{e}-\left( \mu+d_{h}+m \right)H_{i}, \end{aligned} \right.$$

$$\left( 0.2 \right)$$

3. The transmission model with time-dependent control

$$\begin{aligned} &\frac{dH_{s}}{dt}=A_{h}-\left( 1-p\left( t \right) \right)\beta_{1}EH_{s}+\mu H_{i}-d_{h}H_{s}, \\ &\frac{dH_{e}}{dt}=\left( 1-p\left( t \right) \right)\beta_{1}EH_{s}-\nu H_{e}-d_{h}H_{e}, \\ &\frac{dH_{i}}{dt}=\nu H_{e}-\left( \mu+d_{h}+m \right)H_{i}, \\ &\frac{dL_{s}}{dt}=A_{l}-\beta_{2}EL_{s}-\omega\left( t \right)L_{s}+\delta V-\left( \epsilon+d_{l} \right)L_{s}, \\ &\frac{dV}{dt}=\omega\left( t \right)L_{s}-\delta V-\left( \epsilon+d_{l} \right)V, \\ &\frac{dL_{i}}{dt}=\beta_{2}EL_{s}-\left( \epsilon+d_{l} \right)L_{i}, \\ &\frac{dD_{1s}}{dt}=A_{d1}-q\epsilon\beta_{3}L_{i}D_{1s}+\gamma\left( t \right)D_{1i}-dD_{1s}, \\ &\frac{dD_{1i}}{dt}=q\epsilon\beta_{3}L_{i}D_{1s}-\gamma\left( t \right)D_{1i}-dD_{1i}, \\ &\frac{dD_{2s}}{dt}=b_{d2}N_{d2}-\left( 1-q \right)\epsilon\beta_{4}L_{i}D_{2s}-\theta\left( t \right)D_{2s}-\frac{{(b}_{d2}-d{)N}_{d2}}{K_{d2}}D_{2s}-dD_{2s}, \\ &\frac{dD_{2i}}{dt}=\left( 1-q \right)\epsilon\beta_{4}L_{i}D_{2s}-\theta\left( t \right)D_{2i}-\frac{\left( b_{d2}-d \right)N_{d2}}{K_{d2}}D_{2i}-dD_{2i}, \\ &\frac{dE}{dt}=F_{1}\left( h,\eta,\phi\right)D_{1i}+F_{2}\left( h,\eta,\phi\right)D_{2i}-\eta_{1}E. \end{aligned}$$

$$\left( 0.3 \right)$$

4. The objective functional defined as

$$J\left( p\left( t \right),\omega\left( t \right),\gamma\left( t \right), \theta\left( t \right) \right) =\int_{0}^{T} (B_{0}H_{i}(t) +B_{1} L_{i}(t)+C_{1}p^{2}(t)+C_{2}\omega^{2}\left( t \right)+C_{3}\gamma^{2}\left( t \right) +C_{4}\theta^{2}(t))dt$$

$$\left( 0.4 \right)$$

**The positive invariant set.** Define

$$\Gamma=\left\{ \left( H_{s},H_{e},H_{i},L_{s},V,L_{i},D_{1s},D_{1i},D_{2s},D_{2i},E \right)\in R^{11+}:H_{s}+H_{e}+H_{i}\leq\frac{A_{h}}{d_{h}}, L_{s}+V+L_{i}\leq\frac{A_{l}}{d_{l}+\epsilon}, D_{1s}+D_{1i}\leq\frac{A_{d1}}{d}, \right.$$

$$\left. D_{2s}+D_{2i}\leq\frac{\left( b_{d2}-d-\theta\right)K_{d2}}{b_{d2}-d}, E\leq\frac{F_{1}\left( h,\eta,\phi\right)A_{d1}}{d\eta_{1}}+F_{2}\left( h,\eta,\phi\right)\left( b_{d2}-d-\theta\right)K_{d2}/[\left( b_{d2}-d \right)\eta_{1}] \right\},$$

then it is not difficult to show that $\Gamma$ is positively invariant with respect to (0.1) and (0.2), such that any solution with positive initial values will ultimately enter $\Gamma$ as$t\to+\infty$.

**The basic reproduction number.** Following the next generation matrix approach in [1], we calculate the basic reproduction number $R_{0}$ based on $E_{dfe}.$ Note that

$\mathcal{F}=\left[ \begin{aligned} \left( 1-p \right)\beta_{1}EH_{s} \\ 0 \\ \beta_{2}EL_{s} \\ q\epsilon\beta_{3}L_{i}D_{1s} \\ \left( 1-q \right)\epsilon\beta_{4}L_{i}D_{2s} \\ F_{1}D_{1i}+F_{2}D_{2i} \end{aligned} \right]$, $\mathcal{V=}\left[ \begin{aligned} \left( v+d_{h} \right)H_{e} \\ -vH_{e}+\left( \mu+d_{h}+m \right)H_{i} \\ \left( \epsilon+d_{l} \right)L_{i} \\ \left( \gamma+d \right)D_{1i} \\ \left( b_{d2}-d \right)N_{d2}D_{2i}/K_{d2}+\left( \theta+d \right)D_{2i} \\ \eta_{1}E \end{aligned} \right].$

Then, the next generation matrix reads

${FV}^{-1}=\left[ \begin{matrix} 0 & 0 & 0 & 0 & 0 & \frac{\left( 1-p \right)\beta_{1}A_{h}}{d_{h}\eta_{1}} \\ 0 & 0 & 0 & 0 & 0 & 0 \\ 0 & 0 & 0 & 0 & 0 & \frac{\beta_{2}\alpha A_{l}}{\left( \epsilon+d_{l} \right)\eta} \\ 0 & 0 & \frac{q\epsilon\beta_{3}A_{d1}}{\left( \epsilon+d_{l} \right)d} & 0 & 0 & 0 \\ 0 & 0 & \frac{\left( 1-q \right)\epsilon\beta_{4}\left( b_{d2}-d-\theta\right)K_{d2}}{\left( \epsilon+d_{l} \right)\left( b_{d2}-d \right)} & 0 & 0 & 0 \\ 0 & 0 & 0 & \frac{F_{1}}{\gamma+d} & \frac{F_{2}}{b_{d2}} & 0 \end{matrix} \right]$.

The basic reproduction number calculated from $\rho({FV}^{-1})$ is

$$R_{0}=\sqrt[3]{\frac{\beta_{2}\bar{\beta}_{3}\alpha A_{l}A_{d1}F_{1}}{\left( \epsilon+d_{l} \right)^{2}\left( \gamma+d \right)d\eta_{1}}+\frac{\beta_{2}\bar{\beta}_{4}\alpha A_{l}\left( b_{d2}-d-\theta\right)K_{d2}F_{2}}{\left( \epsilon+d_{l} \right)^{2}\left( b_{d2}-d \right)b_{d2}\eta_{1}}}.$$

$$\left( 0.5 \right)$$

We done obtain the existence of the domestic dog-drive endemic equilibrium $E_{d}^{*}$ and the endemic equilibrium $E_{e}^{*}$ by solving the right hand side of system (0.1) and (0.2) for $R_{10}>1$ and $R_{0}>1$*,* respectively.

**Proposition 0.1** *If* $R_{0}>1$*, then system* (0.1) *and* (0.2) *have a unique endemic equilibrium* $E_{e}^{*}$*. If* $R_{10}>1$*, then the domestic dog-drive endemic equilibrium* $E_{d}^{*}$*exists.*

*Proof* Note that system (0.2) is independent of system (0.1), we first look for equilibria of system (0.1). By setting the right-hand side of (0.1) equal to zero, we have $E=\left( F_{1}D_{1i}+F_{2}D_{2i} \right)/\eta_{1}$,$L_{s}+V+L_{i}=A_{l}/\left( d_{l}+\epsilon\right)$, $D_{1s}+D_{1i}=A_{d1}/d$ and $D_{2s}+D_{2i}=\left( b_{d2}-d-\theta\right)K_{d2}/(b_{d2}-d)$ or $D_{2s}+D_{2i}=0.$ Since $D_{2s}+D_{2i}$ can take two possible values, the following discussion is divided into two cases.

**Case 1** $D_{2s}+D_{2i}=\left( b_{d2}-d-\theta\right)K_{d2}/\left( b_{d2}-d \right).$

In this case, to find the endemic equilibrium of system (0.1) is equivalent to solve the following equations,

$$b_{1}D_{1i}^{2}+b_{2}D_{1i}D_{2i}-b_{3}D_{1i}-b_{4}D_{2i}=0,$$

$$(0.6)$$

$$c_{2}D_{1i}D_{2i}-c_{3}D_{1i}+c_{4}D_{2i}=0,$$

$$(0.7)$$

where

$b_{1}= \beta_{2}\bar{\beta}_{3}\alpha\frac{A_{l}}{d_{l}+\epsilon}\frac{F_{1}}{\eta_{1}}+\beta_{2}\left( d+\gamma\right)\frac{F_{1}}{\eta_{1}}$*,* $b_{2}= \beta_{2}\bar{\beta}_{3}\alpha\frac{A_{l}}{d_{l}+\epsilon}\frac{F_{2}}{\eta_{1}}+\beta_{2}(d+\gamma)\frac{F_{2}}{\eta_{1}}$,

$b_{3}= \beta_{2}\bar{\beta}_{3}\alpha\frac{A_{l}}{d_{l}+\epsilon}\frac{A_{d1}}{d}\frac{F_{1}}{\eta_{1}}-{(d_{l}+\epsilon)\beta}_{2}(d+\gamma)$, $b_{4}= \beta_{2}\bar{\beta}_{3}\alpha\frac{A_{l}}{d_{l}+\epsilon}\frac{A_{d1}}{d}\frac{F_{2}}{\eta_{1}},$

$c_{2}=\bar{\beta}_{4}\left( d+\gamma\right)-\bar{\beta}_{3}b_{d2}$, $c_{3}=\bar{\beta}_{4}\frac{b_{d2}-d-\theta}{b_{d2}-d}K_{d2}\left( d+\gamma\right)$,$c_{4}=\bar{\beta}_{3}\frac{A_{d1}}{d}b_{d2}.$

Here$b_{1,}b_{2},b_{4},c_{3}, c_{4}$ are obviously positive and $c_{2}>0$ due to the reasonable biological assumption $\beta_{4}\gg\beta_{3}$. By (0.7), one has

$$D_{2i}=\frac{c_{3}D_{1i}}{c_{4}+c_{2}D_{1i}}.$$

$$(0.8)$$

Substituting (0.8) into (0.6) produces

$$D_{1i}\left( -b_{1}c_{2}D_{1i}^{2}+\left( b_{1}c_{4}+b_{2}c_{3}-b_{3}c_{2} \right)D_{1i}+\left( b_{3}c_{4}+b_{4}c_{3} \right) \right)=0.$$

$$(0.9)$$

Then, it just needs to solve the following quadratic equation

$$-b_{1}c_{2}D_{1i}^{2}+\left( b_{1}c_{4}+b_{2}c_{3}-b_{3}c_{2} \right)D_{1i}+\left( b_{3}c_{4}+b_{4}c_{3} \right)=0.$$

$$(0.10)$$

Since $b_{4}c_{3}+b_{3}c_{4}=W(R_{0}^{3}-1)$, we rewrite (0.10) as

$$G_{1}D_{1i}^{2}+G_{2}D_{1i}+ W\left( R_{0}^{3}-1 \right)=0,$$

$$(0.11)$$

where $G_{1}=-b_{1}c_{2}<0, G_{2}=b_{1}c_{4}+b_{2}c_{3}-b_{3}c_{2}, W=\bar{\beta}_{3}A_{d1}\left( d_{l}+\epsilon\right)\left( d+\gamma\right)b_{d2}/d.$ From the definition of $R_{0}$*, b*y (0.11), it follows that, if$R_{0}<1$*, i.e.,*$b_{3}c_{4}+b_{4}c_{3}<0, G_{2}<0$(see Fig. 10(a)), then (0.11) has two negative roots; if$R_{0}=1, i.e., b_{3}c_{4}+b_{4}c_{3}=0, G_{2}<0$*,* then (0.11) has a zero root and a negative root (see Fig. 10(b)); if $R_{0}>1, i.e., b_{3}c_{4}+b_{4}c_{3}>0$*,* then (0.11) has a unique positive root whether $G_{2}<0 or G_{2}>0$ (see Fig. 10(c)).

(a) (b) (c)

Fig. 10 Location of the parabola defined by (0.11). The open circle denotes the negative root or zero root and the solid circle represents the positive root.

We conclude that (0.11) has a unique positive root $D_{1i}^{*}$if and only if $R_{0}>1$. Then, we claim that, when $R_{0}>1$, the system (0.1) has a unique positive solution, whose components read

$$L_{s}^{*}=\alpha\left( \frac{A_{l}}{d_{l}+\epsilon}-L_{i}^{*} \right), L_{i}^{*}=\frac{\left( d+\gamma\right)D_{1i}^{*}}{\bar{\beta}_{3}\left( \frac{A_{d1}}{d}-D_{1i}^{*} \right)},$$

$V^{*}=\left( 1-\alpha\right)\left( \frac{A_{l}}{d_{l}+\epsilon}-L_{i}^{*} \right),$ $D_{1s}^{*}=\frac{A_{d1}}{d}-D_{1i}^{*},$

$$D_{1i}^{*}=\frac{b_{3}c_{2}-b_{1}c_{4}-b_{2}c_{3}+\sqrt{\left( b_{1}c_{4}+b_{2}c_{3}-b_{3}c_{2} \right)^{2}+4b_{1}c_{2}\left( b_{3}c_{4}+b_{4}c_{3} \right)}}{2b_{1}c_{2}},$$

$$D_{2s}^{*}=\frac{b_{d2}-d-\theta}{b_{d2}-d}K_{d2}-D_{2i}^{*}{, D}_{2i}^{*}=\frac{c_{3}D_{1i}^{*}}{c_{4}+c_{2}D_{1i}^{*}}{, E}^{*}=\frac{F_{1}D_{1i}^{*}+F_{2}D_{2i}^{*}}{\eta_{1}}.$$

Set the right-hand-side of (0.2) be zero, substituting $E^{*}$ into the obtained algebraic equations, then we obtain that (0.2) admits a unique positive equilibrium with

$$H_{s}^{*}=\frac{A_{h}\left( \mu+d_{h}+m \right)\left( v+d_{h} \right)}{\left[ \left( \mu+d_{h}+m \right)\left( v+d_{h} \right)-\mu v \right]\left( 1-p \right)\beta_{1}E^{*}+d_{h}(\mu+d_{h}+m)(v+d_{h})},$$

$$H_{e}^{*}=\frac{\left( 1-p \right)\beta_{1}E^{*}H_{s}^{*}}{v+d_{h}}, H_{i}^{*}=\frac{v\left( 1-p \right)\beta_{1}E^{*}H_{s}^{*}}{(\mu+d_{h}+m)(v+d_{h})}.$$

Thus, if $R_{0}>1$, then the system (0.1) and (0.2) have an endemic equilibrium

$$E_{e}^{*}=\left( H_{s}^{*},H_{e}^{*},H_{i}^{*},L_{s}^{*},V^{*},L_{i}^{*},D_{1s}^{*},D_{1i}^{*},D_{2s}^{*},D_{2i}^{*},E^{*} \right).$$

**Case 2** $D_{2s}+ D_{2i}=0.$

When $R_{10}>1$, it is not diffiffifficult to verify that system (0.1) and (0.2) have a domestic dog-drive endemic equilibrium

$E_{d}^{*}=(\tilde{H}_{s},\tilde{H}_{e},\tilde{H}_{i},\tilde{L}_{s},\tilde{V}{,\tilde{L}}_{i},\tilde{D}_{1s},\tilde{D}_{1i},0,0,\tilde{E})$*,*

where

$$\tilde{H}_{s}=\frac{A_{h}\left( \mu+d_{h}+m \right)\left( v+d_{h} \right)}{\left[ \left( \mu+d_{h}+m \right)\left( v+d_{h} \right)-\mu v \right]\left( 1-p \right)\beta_{1}\tilde{E}+d_{h}(\mu+d_{h}+m)(v+d_{h})},$$

$$\tilde{H}_{e}=\frac{\left( 1-p \right)\beta_{1}\tilde{E}\tilde{H}_{s}}{v+d_{h}}, \tilde{H}_{i}=\frac{v\left( 1-p \right)\beta_{1}\tilde{E}\tilde{H}_{s}}{\left( \mu+d_{h}+m \right)\left( v+d_{h} \right)}, \tilde{L}_{s}=\alpha\left( \frac{A_{l}}{d_{l}+\epsilon}-\tilde{L}_{i} \right),$$

$\tilde{L}_{i}=\left( d+\gamma\right)\frac{\tilde{D}_{1i}}{\bar{\beta}_{3}({A_{d1}}/d-\tilde{D}_{1i})},$ $\tilde{V}=\left( 1-\alpha\right)\left( \frac{A_{l}}{d_{l}+\epsilon}-\tilde{L}_{i} \right),$ $\tilde{D}_{1s}=\frac{A_{d1}}{d}-\tilde{D}_{1i},$

$$\tilde{D}_{1i}=\frac{\left( \epsilon+d_{l} \right)^{2}\left( \gamma+d \right)\eta_{1}\left( R_{10}^{3}-1 \right)}{\beta_{2}F_{1}\alpha\left( A_{l}\bar{\beta}_{3}+\left( \gamma+d \right)\left( d_{l}+\epsilon\right) \right)}, \tilde{E}=\frac{F_{1}\tilde{D}_{1i}}{\eta_{1}}.$$

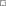


In addition, we prove that the trivial equilibrium $E_{df0}$ is always unstable, the DFE $E_{dfe}$ is globally asymptotically stable if$R_{0}<1$ and it is unstable when $R_{0}>1$. Next, we show the global stability of the endemic equilibrium (EE) $E_{e}^{*}$ using the method in [2, 3]. The results are established as followed.

**Proposition 0.2** $E_{df0}$ *is always unstable.*

*Proof* For the equilibrium $E_{df0}$, one can verify that the characteristic equation is

$$\Phi_{1}\left( \lambda\right):=\left( \lambda+d_{h} \right)\left( \lambda+\mu+d_{h}+m \right)\left( \lambda+v+d_{h} \right)\left( \lambda+d \right)\left( \lambda+d+\theta\right)$$

$\left( \lambda-{(b}_{d2}-d-\theta) \right)\left( \lambda+\epsilon+d_{l} \right)\left( \lambda+\omega+\delta+\epsilon+d_{l} \right)$

$$\left( \left( \lambda+\epsilon+d_{l} \right)\left( \lambda+d+\gamma\right)\left( \lambda+\eta\right)-\beta_{2}\bar{\beta}_{3}\frac{A_{l}A_{d1}}{\left( d_{l}+\epsilon\right)d}F_{1} \right)=0.$$

It is obvious that$\lambda=b_{d2}-d-\theta>0$ is one of its eigenvalues. Hence, by Routh-Hurwitz criteria, $E_{df0}$ is unstable.


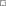


**Proposition 0.3** $E_{dfe}$*is locally asymptotically stable if* $R_{0}<1$ *and* $E_{dfe}$ *is unstable when* $R_{0}>1.$

*Proof* We use Routh-Hurwitz criteria to prove the proposition. The characteristic equation associated with $E_{dfe}$ can be written as

$$\Phi\left( \lambda\right):=\left( \lambda+d_{h} \right)\left( \lambda+\mu+d_{h}+m \right)\left( \lambda+v+d_{h} \right)\left( \lambda+d \right)\left( \lambda+b_{d2}-d-\theta\right)$$

$$\times(\lambda+d)(\lambda+\epsilon+d_{l})(\lambda+\omega+\delta+\epsilon+d_{l})$$

$$\times\left( \lambda+d+\theta\right)\left( \lambda^{4}+a_{1}\lambda^{3}+a_{2}\lambda^{2}+a_{3}\lambda+a_{4} \right)=0.$$

Except for the negative eigenvalues $-d_{h}$*,* $-(\mu+d_{h}+m)$*,* $-\left( b_{d2}-d-\theta\right),-d, -(\epsilon+d_{l}), -\left( \omega+\delta+\epsilon+d_{l} \right)\mathrm{and}$ $-\left( d+\theta\right),$the rest eigenvalues satisfy

$$\Phi_{2}\left( \lambda\right):=\lambda^{4}+a_{1}\lambda^{3}+a_{2}\lambda^{2}+a_{3}\lambda+a_{4}=0,$$

$$(0.12)$$

where

$$a_{1}=\epsilon+d_{l}+d+\gamma+b_{d2}+\eta_{1},$$

$$a_{2}=\eta_{1}\left( d+\gamma\right)+\left( b_{d2}+\epsilon+d_{l} \right)\left( d+\gamma+\eta_{1} \right)+b_{d2}(\epsilon+d_{l}),$$

$a_{3}=\left( 1-R_{10}^{3} \right)\left( \epsilon+d_{l} \right)\left( d+\gamma\right)\eta_{1}+\left( 1-R_{20}^{3} \right)\left( \epsilon+d_{l} \right)b_{d2}\eta_{1}$

$$+b_{d2}\eta_{1}\left( d+\gamma\right)+b_{d2}\left( \epsilon+d_{l} \right)\left( d+\gamma\right),$$

$$a_{4}=\left( 1-R_{0}^{3} \right)\left( \epsilon+d_{l} \right)\left( d+\gamma\right)b_{d2}\eta_{1}.$$

If $R_{0}<1$, we have

$$H_{1}:=a_{1}>0,H_{2}:=a_{1}a_{2}-a_{3}>0,H_{3}:=a_{3}\left( a_{1}a_{2}-a_{3} \right)-a_{1}^{2}a_{4}>0,$$

$$H_{4}:=\left( 1-R_{0}^{3} \right)H_{3}>0.$$

According to the Routh-Hurwitz criteria, all roots of $\Phi_{2}(\lambda)=0$ have negative real parts. Thus, the DFE $E_{dfe}$ is locally asymptotically stable. If $R_{0}>1$, then $a_{4}<0$, which yields that the equation has at least one positive root. Hence, $E_{dfe}$ is unstable. The proof is complete.


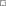


**Theorem 0.1**  *If* $R_{0}\leq1$*, then* $E_{dfe}$ *is globally asymptotically stable.*

*Proof* Consider the subsystem (0.1) and let$(L_{s}(t), V(t), L_{i}(t), D1s(t), D_{1s}\left( t \right) {,D}_{1i}(t), D_{2s}(t),$

$D_{2i}(t), E(t))$be any solution of system (0.1) in $\Gamma$, then, for any $t \geq0$, we have

$L_{s}\left( t \right)\leq\frac{\alpha A_{l}}{d_{l}+\epsilon},$ $D_{1s}\left( t \right)\leq\frac{A_{d1}}{d},$ $D_{2s}\leq\frac{\left( b_{d2}-d-\theta\right)K_{d2}}{b_{d2}-d}.$

Consider a Lyapunov function defined by

$$L=\left( L_{i},D_{1i},D_{2i},E \right)=\eta_{1}L_{i}+\frac{\beta_{2}\alpha A_{l}F_{1}}{\left( d_{l}+\epsilon\right)\left( d+\gamma\right)}D_{1i}+\frac{\beta_{2}\alpha A_{l}F_{2}}{\left( d_{l}+\epsilon\right)b_{d2}}D_{2i}+\frac{\beta_{2}\alpha A_{l}}{d_{l}+\epsilon}E.$$

Differentiating *L* along the solutions of system (0.1) leads to

$$\frac{dL}{dt}=\eta_{1}\beta_{2}EL_{s}-\eta_{1}\left( d_{l}+\epsilon\right)L_{i}+\frac{\beta_{2}\alpha A_{l}F_{1}}{\left( d_{l}+\epsilon\right)\left( d+\gamma\right)}\bar{\beta}_{3}L_{i}D_{1s}-\frac{\beta_{2}\alpha A_{l}F_{1}}{d_{l}+\epsilon}D_{1i}$$

$$+\frac{\beta_{2}\alpha A_{l}F_{2}}{\left( d_{l}+\epsilon\right)b_{d2}}{\bar{\beta}_{4}L_{i}D}_{2s}-\frac{\beta_{2}\alpha A_{l}F_{2}}{\left( d_{l}+\epsilon\right)b_{d2}}\left[ \left( \theta+d \right)+\frac{\left( b_{d2}-d \right)N_{d2}}{K_{d2}} \right]D_{2i}+\frac{\beta_{2}\alpha A_{l}F_{1}}{d_{l}+\epsilon}D_{1i}+\frac{\beta_{2}\alpha A_{l}F_{2}}{d_{l}+\epsilon}D_{2i}-\frac{\beta_{2}\alpha A_{l}}{d_{l}+\epsilon}\eta_{1}E$$

$$\leq\frac{\beta_{2}\alpha A_{l}}{d_{l}+\epsilon}\eta_{1}E-\eta_{1}\left( d_{l}+\epsilon\right)L_{i}+\frac{\beta_{2}\bar{\beta}_{3}\alpha A_{l}{A_{d1}F}_{1}}{\left( d_{l}+\epsilon\right)\left( d+\gamma\right)d}L_{i}-\frac{\beta_{2}\alpha A_{l}F_{2}}{d_{l}+\epsilon}D_{2i}+\frac{\beta_{2}\bar{\beta}_{4}\alpha A_{l}{\left( b_{d2}-d-\theta\right)F}_{1}}{\left( d_{l}+\epsilon\right)\left( b_{d2}-d \right)b_{d2}}L_{i}+\frac{\beta_{2}\alpha A_{l}F_{2}}{d_{l}+\epsilon}D_{2i}-\frac{\beta_{2}\alpha A_{l}}{d_{l}+\epsilon}\eta_{1}$$

$$=\left[ \frac{\beta_{2}\bar{\beta}_{3}\alpha A_{l}{A_{d1}F}_{1}}{\left( d_{l}+\epsilon\right)\left( d+\gamma\right)d}+\frac{\beta_{2}\bar{\beta}_{4}\alpha A_{l}{\left( b_{d2}-d-\theta\right)F}_{1}}{\left( d_{l}+\epsilon\right)\left( b_{d2}-d \right)b_{d2}}-\eta_{1}(d_{l}+\epsilon) \right]L_{i}=\eta_{1}\left( d_{l}+\epsilon\right)\left( R_{0}^{3}-1 \right)L_{i}.$$

Thus, if $R_{0}\leq1$, then $dL/dt\leq0$ and $dL/dt=0$ if and only if $L_{i}=0$. It is not difficult to verify that ${(L}_{s}^{0},V^{0},0,D_{1s}^{0},0,D_{2s}^{0},0,0)$ is the only invariant set of system (0.1).

Hence, by LaSalle’s Invariant Principle [4], ${(L}_{s}^{0},V^{0},0,D_{1s}^{0},0,D_{2s}^{0},0,0)$ is globally asymptotically stable.

Now we consider the subsystem (0.2). Since $E(t)\to0$ as $t\to\infty$*,* one can obtain that $H_{s}(t)\to0$, $H_{e}(t)\to0$, and $H_{i}(t)\to0$as $t\to\infty$. Hence $(A_{h}/d_{h}, 0, 0)$ is attractive with respect to system (0.2). Thus, based on the theory of asymptotic autonomous systems [2], the DFE of system (0.1) and (0.2) is globally asymptotically stable when $R_{0}\leq1$.


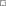


**Theorem 0.2**  *If* $R_{10}>1$*, then* $E_{d}^{*}$ *is unstable.*

*Proof* The characteristic equation of the Jacobian matrix at $E_{d}^{*}$ is

$$\Phi_{3}\left( \lambda\right):=\left[ \left( \lambda+\left( 1-p \right)\beta_{1}\tilde{E}+d_{h} \right)\left( \lambda+\gamma+d_{h} \right)\left( \lambda+\mu+d_{h}+m \right)-\left( 1-p \right)\beta_{1}\tilde{E}\mu v \right]$$

$$\times\left[ \left( \lambda-b_{d2}+d+\theta+\bar{\beta}_{4}\tilde{L}_{i} \right)\left( \lambda+d+\theta\right)-b_{d2}\bar{\beta}_{4}\tilde{L}_{i} \right]$$

$$\times\left\{ \left[ \left( \left( \lambda+\delta+\epsilon+d_{l} \right)\left( \lambda+\beta_{2}\tilde{E}+\omega+\epsilon+d_{l} \right)-\omega\delta\right)\left( \lambda+\epsilon+d_{l} \right)\left( \lambda+\eta_{1} \right) \right. \right.$$

$$\left. \left( \lambda+\bar{\beta}_{3}\tilde{\beta}_{3}+d\left( \lambda+\gamma+d \right)-\bar{\beta}_{3}\tilde{L}_{i}\gamma\right) \right]-\bar{\beta}_{2}\tilde{L_{s}}F_{1}\left( \left( \lambda+\bar{\beta}_{3}\tilde{L}_{i}+d \right)\bar{\beta}_{3}\tilde{D}_{1s}-\bar{\beta}_{3}\tilde{L}_{i}\left( \bar{\beta}_{3}\tilde{D}_{1s}+d \right) \right)$$

$$\left( \left( \lambda+\delta+\epsilon+d_{l} \right)\left( \lambda+\beta_{2}\tilde{E}+\omega+\epsilon+d_{l} \right)-\omega\delta\right)$$

$$\left. -\bar{\beta}_{2}\tilde{L}_{i}F_{1}\left( \left( \lambda+\bar{\beta}_{3}\tilde{L}_{i}+d \right)\bar{\beta}_{3}\tilde{D}_{1s}-\bar{\beta}_{3}\tilde{L}_{i}\left( \bar{\beta}_{3}\tilde{D}_{1s}+d \right) \right) \right\}$$

$$=\left( \lambda^{3}+b_{2}\lambda^{2}+b_{1}\lambda+b_{0} \right)\left( \lambda^{2}+c_{1}\lambda+c_{0} \right)$$

$$\left( \lambda^{6}+g_{5}\lambda^{5}+g_{4}\lambda^{4}+g_{3}\lambda^{3}+g_{2}\lambda^{2}+g_{1}\lambda^{1}+g_{0} \right)=0,$$

where *λ* denotes the eigenvalue and we just discuss the part

$$\Phi_{3}^{2}\left( \lambda\right)=\lambda^{2}+c_{1}\lambda+c_{0}.$$

Note that

$c_{1}=2\left( d+\theta\right)+\bar{\beta}_{4}\tilde{L}_{i}-b_{d2}$*,* $c_{0}=\left( d+\theta-b_{d2} \right)\left( \bar{\beta}_{4}\tilde{L}_{i}+d+\theta\right)<0,$

since $b_{d2}-d-\theta>0$. Then, there must be a positive root of $\Phi_{3}\left( \lambda\right)=0$, i.e., there is a positive eigenvalue of $J(E_{d}^{*})$. Hence, by Routh-Hurwitz criteria, $E_{d}^{*}$ is unstable.


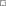


**Theorem 0.3**  *If* $R_{0}>1$*, then* $E_{e}^{*}$ *is globally asymptotically stable.*

*Proof* First, we investigate the globally asymptotic stability of the positive equilibrium $E_{e}^{*}$ of system (0.1). Note that, as $t\to\infty$, we have

$$L_{s}\left( t \right)+V\left( t \right)+L_{i}\left( t \right)\to\frac{A_{l}}{d_{l}+\epsilon}, D_{1s}+D_{1i}\left( t \right)\to\frac{A_{d1}}{d},$$

$$D_{2s}\left( t \right)+D_{2i}\left( t \right)\to\frac{\left( b_{d2}-d-\theta\right)K_{d2}}{b_{d2}-d}.$$

When $R_{0}>1$, the limiting system of system (0.1) is

$$\left\{ \begin{aligned} &\frac{dL_{i}}{dt}=\beta_{2}E\alpha\left( \frac{A_{l}}{d_{l}+\epsilon}-L_{i} \right)-\epsilon L_{i}-d_{l}L_{i}, \\ &\frac{dD_{1i}}{dt}=\bar{\beta}_{3}L_{i}\left( \frac{A_{d1}}{d}-D_{1i} \right)-\gamma D_{1i}-dD_{1i}, \\ &\frac{dD_{2i}}{dt}=\bar{\beta}_{4}L_{i}\left( \frac{\left( b_{d2}-d-\theta\right)K_{d2}}{b_{d2}-d}-D_{2i} \right)-b_{d2}D_{2i}, \\ &\frac{dE}{dt}=F_{1}D_{1i}+F_{2}D_{2i}-\eta_{1}E. \end{aligned} \right.$$

$$(0.13)$$

Since $\Gamma$ is positively invariant with respect to system (0.1) and (0.2), the dynamics of system (0.16) can be explored on the following restricted region $Ω$, where

$$\Omega=\{\left( L_{i}.D_{1i},D_{2i},E \right)\in R_{+}^{4}:L_{i}\leq\frac{\alpha A_{l}}{d_{l}+\epsilon},D_{1i}\leq\frac{A_{d1}}{d},D_{2i}\leq\frac{\left( b_{d2}-d-\theta\right)K_{d2}}{b_{d2}-d},$$

$$E\leq\frac{F_{1}A_{d1}}{d\eta_{1}}+F_{2}\left( b_{d2}-d-\theta\right)K_{d2}/[(b_{d2}-d)\eta_{1}]\}.$$

Next, we explore the globally asymptotic stability of the endemic equilibrium of system (0.1) by studying the limiting system (0.13). Motivated by the method in [3], define

$$g\left( v \right)=\left( \begin{aligned} g_{1}\left( v_{1},v_{2},v_{3}.v_{4} \right) \\ g_{2}\left( v_{1},v_{2},v_{3}.v_{4} \right) \\ g_{3}\left( v_{1},v_{2},v_{3}.v_{4} \right) \\ g_{4}\left( v_{1},v_{2},v_{3}.v_{4} \right) \end{aligned} \right)=\left( \begin{aligned} \beta_{2}\alpha\left( \frac{A_{l}}{d_{l}+\epsilon}-v_{1} \right)v_{4}-\left( d_{l}+\epsilon\right)v_{1} \\ \bar{\beta}_{3}\left( \frac{A_{d1}}{d}-v_{2} \right)v_{1}-\left( d+\gamma\right)v_{2} \\ \bar{\beta}_{4}\left( \frac{\left( b_{d2}-d-\theta\right)K_{d2}}{b_{d2}-d}-v_{3} \right)v_{1}-b_{d2}v_{3} \\ F_{1}v_{2}+F_{2}v_{3}-\eta_{1}v_{4} \end{aligned} \right),$$

then $g:R_{+}^{4}\to R_{+}^{4}$ is a continuously differentiable mapping. Obviously,

$g(0) = 0, g_{i}(v)\geq0$ for all $v\inΩ$*,* with $v_{i}=0, i=1, 2, 3, 4.$

Since

$$\frac{\partial g_{i}}{\partial v_{j}}\geq0 \left( i\neq j \right),v\inΩ,$$

so *g* is cooperative on Ω.

For every $k\in(0, 1)$ and $v\inΩ$, we have

$$g_{i}\left( kv_{1},{kv}_{2},kv_{3},kv_{4} \right)=\beta_{2}\alpha\left( \frac{A_{l}}{d_{l}+\epsilon}-kv_{1} \right)kv_{4}-\left( d_{l}+\epsilon\right)kv_{1}$$

$$\geq\beta_{2}\alpha\left( \frac{A_{l}}{d_{l}+\epsilon}-v_{1} \right)kv_{4}-\left( d_{l}+\epsilon\right)kv_{1}=kg_{1}\left( v_{1},v_{2},v_{3}.v_{4} \right).$$

Using the same argument, we find that

$$g_{i}\left( kv_{1},{kv}_{2},kv_{3},kv_{4} \right)\geq kg_{i}\left( v_{1},v_{2},v_{3}.v_{4} \right), i=2,3,4.$$

Therefore, *g* is strictly sublinear on Ω. A straightforward calculation of $D_{g}(v)$ gives

$$D_{g}\left( v \right)=\left[ \begin{matrix} -\beta_{2}\alpha v_{4}-(d_{l}+\epsilon) & 0 & 0 & \beta_{2}\alpha\left( \frac{A_{l}}{d_{l}+\epsilon}-v_{1} \right) \\ \bar{\beta}_{3}\left( \frac{A_{d1}}{d}-v_{2} \right) & -\bar{\beta}_{3}v_{1}-(d+\gamma) & 0 & 0 \\ \bar{\beta}_{4}\left( \frac{r_{d2}K_{d2}}{b_{d2}-d}-v_{3} \right) & 0 & -\bar{\beta}_{4}v_{1}-b_{d2} & 0 \\ 0 & F_{1} & F_{2} & -\eta_{1} \end{matrix} \right],$$

where $r_{d2}=b_{d2}-d-\theta$*.* Obviously, $|D_{g}(v)|\neq0$*,* $D_{g}(v)$ is irreducible on $v\inΩ$. Since

$$D_{g}\left( 0 \right)=\left[ \begin{matrix} -(d_{l}+\epsilon) & 0 & 0 & \beta_{2}\alpha\frac{A_{l}}{d_{l}+\epsilon} \\ \bar{\beta}_{3}\frac{A_{d1}}{d} & -(d+\gamma) & 0 & 0 \\ \bar{\beta}_{4}\frac{{(b_{d2}-d-\theta)K}_{d2}}{b_{d2}-d} & 0 & -b_{d2} & 0 \\ 0 & F_{1} & F_{2} & -\eta_{1} \end{matrix} \right],$$

the characteristic equation of $D_{g}(0)$is $\Phi_{2}(\lambda)=0$, which is defined by (0.12). Then, if $R_{0}>1$, one has

$$s\left( D_{g}\left( 0 \right) \right):=\max\left\{ Re\lambda:\Phi\left( \lambda\right) \right\}>0.$$

By Corollary 3.2 in [3], we conclude that the equilibrium $\left( L_{i}^{*},D_{1i}^{*}, D_{2i}^{*},E^{*} \right)$of system (0.13) is globally asymptotically stable. Similarly, one can prove that $L_{s}^{*},V^{*},L_{i}^{*},D_{1s}^{*},D_{1i}^{*},D_{2s}^{*},D_{2i}^{*},E^{*}$ is globally attractive for system (0.1).

Moreover, when $R_{0}>1$, the limiting system of system (0.2) becomes

$$\left\{ \begin{aligned} &\frac{dH_{s}}{dt}=A_{h}-\left( 1-p \right)\beta_{1}E^{*}H_{s}+\mu H_{i}-d_{h}H_{s}, \\ &\frac{dH_{e}}{dt}=\left( 1-p \right)\beta_{1}E^{*}H_{s}+vH_{e}-d_{h}H_{e}, \\ &\frac{dH_{i}}{dt}=vH_{e}-\left( \mu+m+d_{h} \right)H_{i}. \end{aligned} \right.$$

$$(0.14)$$

It can be verified that the equilibrium $\left( H_{s}^{*}, H_{e}^{*},H_{i}^{*} \right)$of system (0.14) is globally asymptotically stable. According to the theory of asymptotic autonomous systems [2], we conclude that $E_{e}^{*}$ is globally asymptotically stable.


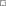


**Theorem 0.4**  *The optimal control of* (0.3) *is characterized by*

$$p^{*}\left( t \right)=\min\left\{ p_{max},max\left\{ 0,\frac{\left( \lambda_{2}-\lambda_{1} \right)\beta_{1}E\left( t \right)H_{s}(t)}{2C_{1}} \right\} \right\},$$

$$\omega^{*}\left( t \right)=\min\left\{ \omega_{max},max\left\{ 0,\frac{\left( \lambda_{4}-\lambda_{5} \right)L_{s}(t)}{2C_{2}} \right\} \right\},$$

$$\gamma^{*}\left( t \right)=\min\left\{ \gamma_{max},max\left\{ 0,\frac{\left( \lambda_{8}-\lambda_{7} \right)D_{1i}\left( t \right)}{2C_{3}} \right\} \right\},$$

$$\theta^{*}(t)=\min\left\{ \theta_{max},max\left\{ 0,\frac{\lambda_{9}D_{2s}\left( t \right)+\lambda_{10}D_{2i}(t)}{2C_{4}} \right\} \right\},$$

$$(0.15)$$

*where*$\lambda_{i},i=1, 2, . . . , 11$ *are the adjoint variables satisfying the adjoint equations* (0.16) *and the transversality conditions.*

*Proof* By Potryagin’s Maximum Principle, as in [5, 6], to find the optimal control of (0.4) is equivalent to minimize the following Hamiltonian

$$H=B_{0}H_{i}\left( t \right)+B_{1}L_{i}\left( t \right)+C_{1}p\left( t \right)^{2}+C_{2}\omega\left( t \right)^{2}+C_{3}\gamma\left( t \right)^{2}+C_{4}\theta\left( t \right)^{2}+\sum_{i=1}^{11} \lambda_{i}f_{i},$$

where $f_{i},i=1, 2, . . . , 11$denote the right hand sides of system (0.3) and $\lambda_{i},i=1, 2, . . . , 11$are the adjoint variables satisfying the following adjoint equations

$$\lambda_{1}^{'}=-\frac{\partial H}{\partial H_{s}}=\lambda_{1}\left( \left( 1-p\left( t \right) \right)\beta_{1}E\left( t \right)+d_{h} \right)-\lambda_{2}\left( 1-p\left( t \right) \right)\beta_{1}E\left( t \right),$$

$$\lambda_{2}^{'}=-\frac{\partial H}{\partial H_{e}}=\lambda_{2}\left( v+d_{h} \right)-\lambda_{3}v,$$

$$\lambda_{3}^{'}=-\frac{\partial H}{\partial H_{i}}={-B_{0}-\lambda}_{1}\mu+\lambda_{3}(\mu+d_{h}+m),$$

$$\lambda_{4}^{'}=-\frac{\partial H}{\partial L_{s}}=\lambda_{4}\left( \beta_{2}E\left( t \right)+\omega\left( t \right)+\epsilon+d_{l} \right)-\lambda_{5}\omega\left( t \right)-\lambda_{6}\beta_{2}E\left( t \right),$$

$$\lambda_{5}^{'}=-\frac{\partial H}{\partial V}={-\lambda}_{4}\delta+\lambda_{5}(\delta+\epsilon+d_{l}),$$

$$\lambda_{6}^{'}=-\frac{\partial H}{\partial L_{i}}=-B_{1}+\lambda_{6}\left( \epsilon+d_{l} \right)+\lambda_{7}q\epsilon\beta_{3}D_{1s}\left( t \right)-\lambda_{8}q\epsilon\beta_{3}D_{1s}\left( t \right)+\lambda_{9}\left( 1-q \right)\epsilon\beta_{4}D_{2s}\left( t \right)-\lambda_{10}\left( 1-q \right)\epsilon\beta_{4}D_{2s}\left( t \right),$$

$$\lambda_{7}^{'}=-\frac{\partial H}{\partial D_{1s}}=\lambda_{7}\left( q\epsilon\beta_{3}L_{i}\left( t \right)+d \right)-\lambda_{8}q\epsilon\beta_{3}L_{i}\left( t \right),$$

$$\lambda_{8}^{'}=-\frac{\partial H}{\partial D_{1i}}=-\lambda_{7}\gamma\left( t \right)+\lambda_{8}\left( \gamma\left( t \right)+d \right)-\lambda_{11}F_{1}(h,\eta,\phi),$$

$$\lambda_{9}^{'}=-\frac{\partial H}{\partial D_{2s}}=\lambda_{9}\left( \theta\left( t \right)+d-b_{d2}+\left( 1-q \right)\epsilon\beta_{4}L_{i}\left( t \right)+\frac{b_{d2}-d}{K_{d2}}\left( 2D_{2s}\left( t \right)+D_{2i}\left( t \right) \right) \right)+\lambda_{10}\left( \frac{b_{d2}-d}{K_{d2}}D_{2i}-(1-q)\epsilon\beta_{4}L_{i}\left( t \right) \right),$$

$$\lambda_{10}^{'}=-\frac{\partial H}{\partial D_{2i}}=\lambda_{9}\left( \frac{b_{d2}-d}{K_{d2}}D_{2s}-b_{d2} \right)+\lambda_{10}\left( \frac{b_{d2}-d}{K_{d2}}\left( D_{2s}\left( t \right)+2D_{2i}\left( t \right) \right)+\theta\left( t \right)+d \right)-\lambda_{11}F_{2}(h,\eta,\phi),$$

$$\lambda_{11}^{'}=-\frac{\partial H}{\partial E}=\lambda_{1}\left( 1-p\left( t \right) \right)\beta_{1}H_{s}\left( t \right)-\lambda_{2}\left( 1-p\left( t \right) \right)\beta_{1}H_{s}\left( t \right)+\lambda_{4}\beta_{2}L_{s}\left( t \right)-\lambda_{6}\beta_{2}L_{s}\left( t \right)+\lambda_{11}\eta_{1},$$

$$(0.16)$$

and the transversal conditions

$$\lambda_{i}\left( T \right)=0, i=1, 2, . . . , 11.$$

The optimal conditions

$$\frac{\partial H}{\partial p(t)}=2C_{1}p\left( t \right)+\lambda_{1}\beta_{1}E\left( t \right)H_{s}\left( t \right)-\lambda_{2}\beta_{1}E\left( t \right)H_{s}\left( t \right)=0,$$

$$\frac{\partial H}{\partial\omega(t)}=2C_{2}\omega\left( t \right)-\lambda_{4}L_{s}\left( t \right)+\lambda_{5}L_{s}\left( t \right)=0,$$

$$\frac{\partial H}{\partial\gamma(t)}=2C_{3}\gamma\left( t \right)+\lambda_{7}D_{1i}\left( t \right)-\lambda_{8}D_{1i}\left( t \right)=0,$$

$$\frac{\partial H}{\partial\theta(t)}=2C_{4}\theta\left( t \right)-\lambda_{9}D_{2s}\left( t \right)-\lambda_{10}D_{2i}\left( t \right)=0,$$

lead to

$$p^{*}\left( t \right)=\frac{\left( \lambda_{2}-\lambda_{1} \right)\beta_{1}E\left( t \right)H_{s}\left( t \right)}{2C_{1}} , \omega^{*}\left( t \right)=\frac{\left( \lambda_{4}-\lambda_{5} \right)L_{s}\left( t \right)}{2C_{2}},$$

$$\gamma^{*}\left( t \right)=\frac{(\lambda_{8}-\lambda_{7})D_{1i}\left( t \right)}{2C_{3}}, \theta^{*}\left( t \right)=\frac{\lambda_{9}D_{2s}\left( t \right)+\lambda_{10}D_{2i}(t)}{2C_{4}}.$$

Moreover, taking into account the fact that $u^{*} :=(p^{*}(t), \omega^{*}(t), \gamma^{*}(t), \theta^{*}(t))\in U,$ i.e., using the lower and upper bounds of $u^{*}$ , we find that the optimal control $u^{*} :=(p^{*}(t), \omega^{*}(t), \gamma^{*}(t), \theta^{*}(t))\in U$ is well characterized by (0.15).


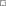


References

1. Van DP den, Watmough J. Reproduction numbers and subthreshold endemic equilibria for compartmental models of disease transmission. Math Biosci. 2002;180(1):29–48.
2. Thieme HR. Convergence results and a Poincare-Bendixson trichotomy for asymptotically autonomous differential equations. J Math Biol. 1992;30(7):755–763.
3. Zhao XQ, Jing ZJ. Global asymptotic behavior in some cooperative systems of functional differential equations. Canad Appl Math Quart. 1996;4(4):421–444.
4. LaSalle JP, Lefschetz S. Stability by Liapunov’s direct method. 1: Academic Press; 1961.
5. Pontryagin V Boltyanskii, Gamkrelize R, Mishchenko E. The mathematical theory of optimal processes. New York: Wiley; 1967.
6. Clayton TJ. Optimal Control of Epidemic Models Involving Rabies and West Nile Viruses. University of Tennessee; 2008.
